# Neurosteroids are reduced in diabetic neuropathy and may be associated with the development of neuropathic pain

**DOI:** 10.12688/f1000research.9034.1

**Published:** 2016-08-05

**Authors:** Stephen R. Humble

**Affiliations:** 1 Department of Anaesthetics and Pain Management, Charing Cross Hospital, Imperial College NHS Healthcare Trust London, London, W6 8RF, UK

**Keywords:** Neuropathic pain, Neurosteroids, diabetes, neuropathy, GABAAR, GABAA receptor, ob ob, db db

## Abstract

Introduction: Peripheral and central sensitisation are implicated in the development of neuropathic pain. Hypersensitivity of pain pathway neurons has been described in animal models of diabetic neuropathy, which is postulated to be related to an imbalance between inhibitory and excitatory signals within the spinal cord. GABAergic neurons within the pain pathway are vital for the transmission of painful stimuli to higher centres. A developmental change in the rate of exponential decay of GABAergic synaptic events has been observed in other types of neurons and this may be associated with fluctuations in endogenous neurosteroid tone.

Methods: The whole-cell patch-clamp technique was used on slices of neural tissue. Electrophysiological recordings were obtained from wild type mice between the ages of 6 and 80 days in the spinal cord, the nucleus reticularis of the thalamus and the cerebral cortex. Recordings were also obtained from mice with diabetic neuropathy (ob/ob and db/db) between the ages of 60 and 80 days. Behavioural experiments were performed to examine mechanical and thermal nociception.

Results: Electrophysiological recordings from cortical pain pathway neurons from mature type-2 diabetic mice revealed that the endogenous neurosteroid tone is reduced compared to control. However, selected neurosteroid compounds had a more pronounced effect on the GABA _A_ receptors of these diabetic mice. ob/ob mice exhibit mechanical hyperalgesia and allodynia, which was reduced by neurosteroids applied exogenously.

Conclusions: The reduced endogenous neurosteroid tone in ob/ob mice may be linked to their hypersensitivity. Neurosteroids may exert analgesic effects in pathological pain states by attempting to restore the physiological GABAergic inhibitory tone.

**Text box 1**

**What's already known about this topic?**

- There is a worldwide obesity diabetes epidemic, causing a huge amount of morbidity including neuropathy and neuropathic pain.
- The mechanisms responsible for the development of neuropathy and neuropathic pain are not well understood.
- Peripheral and central sensitisation are implicated in the development of neuropathic pain with neuroplastic changes occurring at multiple levels of the pain pathway.
- Hypersensitivity of pain pathway neurons has been described in animal models of diabetic neuropathy, which is postulated to be related to an imbalance between inhibitory and excitatory signals within the spinal cord. GABAergic neurons within the pain pathway are vital for the transmission of painful stimuli to higher centres involved in the perception of pain.
- GABA _A_ receptors are an important target for many drugs, and specific endogenous neurosteroids act as potent allosteric modulators of these receptors.
- A developmental change in the rate of exponential decay of GABAergic synaptic events has been observed in other types of neurons and this may be associated with fluctuations in endogenous neurosteroid tone.

**What does this study add?**

- This paper explores potential underlying mechanisms and identifies potential therapeutic targets in order to promote translational work in this field.
- This is the first electrophysiological GABA _A_ receptor characterisation of two models of type-2 diabetes mellitus and reports the discovery of a reduced endogenous GABA-ergic neurosteroid tone that may mediate painful neuropathic hypersensitivity.
- The paper then reports the anti-nociceptive impact of neurosteroids on live mice, thus complementing the electrophysiological data.
- These discoveries may ultimately lead to a new rational avenue of research aimed at understanding and treating painful diabetic neuropathy and the neuropathic mechanisms may be analogous in other neuropathic conditions such as chemotherapy-induced neuropathic pain.

## Introduction

**Figure 1.**
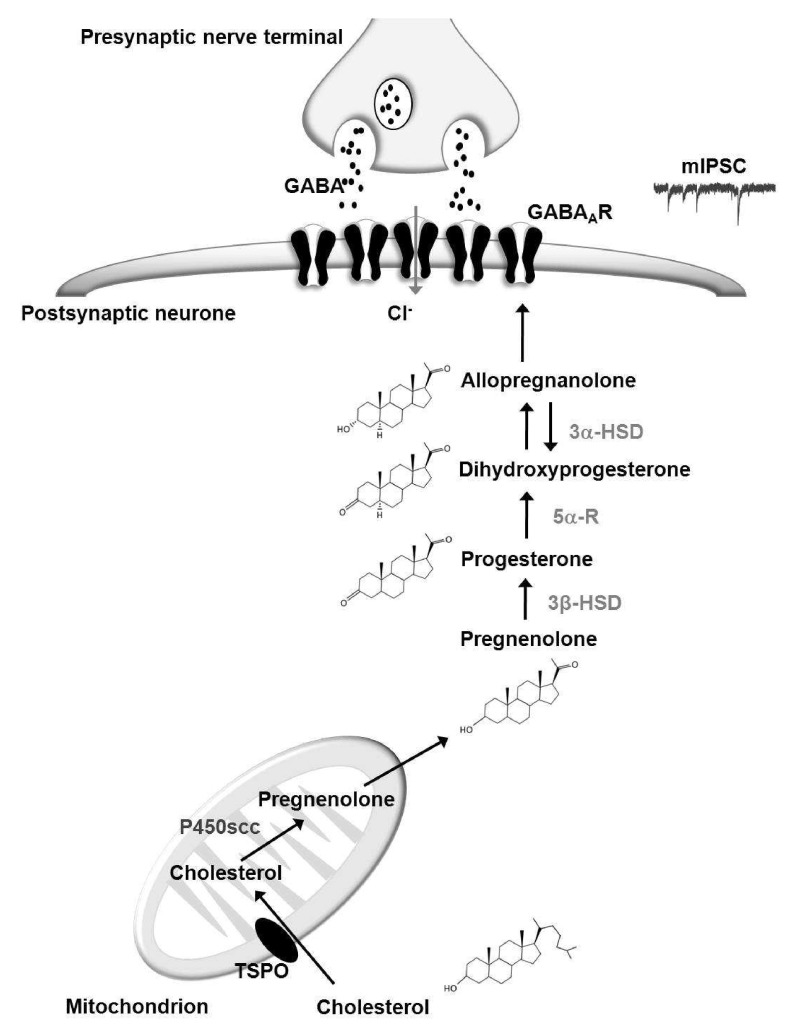
Modulation of the GABA _A_R by endogenous neurosteroids. Cholesterol is taken through the mitochondrial membrane by the translocator protein where it is converted to pregnenolone by the cytochrome P450 side chain cleavage enzyme. Pregnenolone undergoes stepwise enzymatic conversion to other neurosteroid compounds and ultimately allopregnanolone which modulates GABA _A_R function. Neurosteroids may act *via* paracrine or autocrine mechanisms. Postsynaptic GABA _A_Rs are activated by GABA that has been released from vesicles in the presynaptic nerve terminal. GABA induces a conformational change in the GABA _A_R, which ‘opens’ its central pore, allowing the passage of chloride ions. The negatively charged chloride ions typically induce hyperpolarisation of the neuronal membrane, which is associated with neuronal inhibition. Neurosteroids such as allopregnanolone enhance GABA _A_R function and therefore facilitate neural inhibition.

A loss of physiological inhibitory tone is associated with hypersensitivity to painful stimuli such as allodynia and hyperalgesia ( Chen & Pan, 2002; Zeilhofer, 2008). The GABA _A_ receptor (GABA _A_R) is the major inhibitory receptor in the mammalian nervous system and mediates inhibitory tone throughout the pain pathway ( D’Hulst *et al.*, 2009; Johnston, 2005). Reduced GABAergic inhibition within the spinal cord may be implicated in the development of hypersensitivity to nociceptive stimuli ( Munro *et al.*, 2009; von Hehn *et al.*, 2012; Zeilhofer, 2008). Therefore, pharmacological agents that enhance GABA _A_R function could be useful to counteract lost inhibitory tone ( Knabl *et al.*, 2008; Munro *et al.*, 2009). Neurosteroids such as allopregnanolone are potent allosteric modulators of this receptor ( Callachan *et al.*, 1987; Hosie *et al.*, 2006; Figure 1). Indeed, an upregulation in the production of endogenous neurosteroids within the spinal cord in response to peripheral inflammation has been shown to have an analgesic effect. The analgesic effect could be suppressed by the administration of finasteride to inhibit the enzyme 5α-R, which converts progesterone to its more active metabolites ( Poisbeau *et al.*, 2005; Schlichter *et al.*, 2006).

There is a burgeoning obesity and type-2 diabetes epidemic worldwide and the incidence is likely to become even greater ( Danaei *et al.*, 2011). Neurosteroids such as progesterone have been studied for their potentially protective effects for a number of different neuropathologies including stroke, brain and spinal injuries ( Mensah-Nyagan *et al.*, 2009; Stein, 2008). It is possible that fluctuations in endogenous neurosteroid levels may have a pathophysiological role in the development of diabetic neuropathic pain. In mice, diabetic neuropathy develops over weeks, which allows disease progression and the efficacy of interventions to be studied within a relatively a short timeframe ( Cefalu, 2006; Kaplan & Wagner, 2006). The type-2 diabetic *ob/ob* mouse, which has an autosomal recessive nonsense mutation on chromosome 6 causing leptin deficiency develops morbid obesity due to its rapacious appetite and exhibits a predictable and spontaneous neuropathic phenotype ( Drel *et al.*, 2006; Latham *et al.*, 2009; Lindstrom, 2007; Vareniuk *et al.*, 2007). In contrast, the *db/db* mouse has an autosomal recessive mutation of the leptin receptor gene, which prevents leptin from activating its receptor ( Chen *et al.*, 1996; Chung *et al.*, 1996).

Pain has sensory, emotional and cognitive components and therefore multiple areas within the cerebral cortex are involved in generating the experience of pain ( Flor & Bushnell, 2005; Treede *et al.*, 1999). Areas such as the insular, somatosensory, anterior cingulate and prefrontal cortices and the thalamus may be considered as a ‘pain matrix.’ The cerebral cortex is organised into parallel mini-columns of synaptically linked neurons that span part, or all of the six horizontal cortical layers. Mini-columns may be clustered together to constitute functional modules ( Lubke & Feldmeyer, 2007; Mountcastle, 1997). GABAergic inhibitory interneurons are present in all layers of the cortex but are most abundant in layers 2/3 and lower in layer 4 ( Meyer *et al.*, 2011). By improving the understanding at the molecular level it may be possible to identify novel therapeutic targets for painful diabetic neuropathy.

## Methods

### Breeding and housing of mice

All procedures were carried out in compliance with the University of Dundee code of practice following consideration by the University Research Ethics Committee (UREC) and in accordance with Schedule 1 of the Animals (Scientific Procedures) Act 1986 (UK). Home Office Project Licence numbers: PPL 60/4144 and PPL 60/4005. Wellcome Trust Grant number 090667. Wild Type (WT) mice aged less than 2 months were obtained from an in-house colony. C57/Bl6J WT mice aged 2 months were purchased from Charles River, UK while OlaHSD *ob/ob* mice, *db/db* mice and their respective strain-matched WT littermates, all aged 2 months were purchased from Harlan UK and housed in the same in-house colony. All animals were male and were kept under an alternating 12hr light/dark cycle and had *ad libitum* access to food and water.

### Spinal cord dissection technique

Prior to dissection mice were killed instantly by cervical dislocation (Schedule 1), the tissue was then submerged immediately into a bath of ice-cold oxygenated (95% O _2_/5% CO _2_) artificial cerebrospinal fluid (aCSF). The aCSF comprised: 225mM sucrose, 10mM glucose, 10mM MgSO _4_, 26mM NaHCO _3_, 1.25mM NaH _2_PO _4_, 2.5mM KCl, 0.5mM CaCl _2_, 1mM ascorbic acid and 3mM pyruvic acid (total osmolarity ~330mosm/l). The spinal cord was extracted by anterior laminectomy as per Keller *et al.* (2001). The cord was immediately set within agar gel, which was glued to the Leica VT1000S vibratome (Heidelberg, Germany) for slicing. Horizontal, thoracolumbar slices, (300–350μm) were cut and transferred onto a nylon mesh platform within a storage chamber containing oxygenated (95% O _2_/5% CO _2_) artificial extracellular solution (aECS) comprising: 126mM NaCl, 10mM glucose, 2mM MgCl _2_, 26mM NaHCO _3_, 1.25mM NaH _2_PO _4_, 2.95mM KCl, 2mM CaCl _2_, 1mM ascorbic acid, 3mM pyruvic acid and 2mM kynurenic acid (Total measured osmolarity ~310mOsm/l). Slices were stored at room temperature for at least one hour before being used to obtain electrophysiological recordings.

### Brain slicing technique

Brain tissue was also obtained following the cervical dislocation method (Schedule 1) and submerged in aCSF as above. For all nucleus reticularis (nRT) preparations the aCSF solution was the same as that described above for spinal cord but the sucrose concentration was increased to 234mM giving a total osmolarity of ~340mOsm/l as this improved the condition of the slices. For cortical preparations in mice below 2 months of age the aCSF was the same as for the spinal cord. Neuronal viability deteriorates with increasing age and for mice above the age of 2 months, including all *ob/ob* and *db/db*, a different solution was required to optimise the condition of the slices as previously described by Maguire *et al.* (2014). This consisted of 140mM potassium gluconate, 10mM HEPES, 15mM sodium gluconate, 0.2mM EGTA, 4mM NaCl, 1mM ascorbic acid and 3mM pyruvic acid. Sodium hydroxide solution was then added to bring the pH up to 7.2. The brain was removed carefully from the skull and slices were obtained using a Vibratome series 1000 PLUS Sectioning System (Intracell, Royston, Hertfordshire, UK). Cortical slices were cut in the coronal plane and nRT slices in the horizontal plane. Slice thickness was greatest for the youngest mice and least for the oldest mice (250–350μM). Slices were transferred immediately to a storage chamber as previously described.

### Electrophysiology

Subsequently, the slices were transferred to a recording chamber under an Olympus BX51WI fixed-stage upright microscope. An infrared differential interference contrast disc and a water immersion objective (× 40) were used for visualisation of neurons within the dorsal horn of the spinal cord, the nucleus reticularis of the thalamus and the somatosensory area of the cortex respectively. The chamber was perfused continuously with oxygenated artificial extracellular solution (aECS). Slices were held in position using a small grid. Whole-cell patch-clamp recordings were obtained using an Axopatch 200B amplifier and all recordings were made at a holding potential of -60mV and at a temperature of 35°C. The extracellular solution contained kynurenic acid (2mM), strychnine (0.5μM) and tetrodotoxin (0.5μM) to antagonise ionotropic glutamate, glycine receptors and voltage-gated sodium channels respectively. Patch electrodes were prepared from thick walled borosilicate glass capillaries (Garner Glass Co., Claremont, CA, USA). Such electrodes had an open-tip resistance of ~4MΩ when filled with intracellular solution containing: 135mM CsCl, 10mM HEPES, 10mM EGTA, 1mM CaCl _2_, 1mM MgCl _2_, 2mM Mg-ATP, 5mM QX-314, pH 7.2, titrated with CsOH. The measured osmolarity was 310mOsmol/l. Typically, the mean whole-cell capacitance was 5–15pF. Series resistance was compensated for by up to 80% and recordings were considered invalid where the series resistance changed by more than 20% or if it exceeded 15MΩ. A 2 kHz frequency filter was used for all recordings and analysis of each cell was performed offline.

### Data analysis

The Strathclyde Electrophysiology Software, WinEDR and WinWCP, (Dr J Dempster, University of Strathclyde, Glasgow, UK) was used for the analysis of recordings. Only recordings that met specific quality criteria were included in the analysis. Using an algorithmic detection protocol, miniature inhibitory postsynaptic currents (mIPSCs) were detected with an amplitude threshold of at least -5pA and duration > 2ms. Each individual mIPSC was then inspected visually to ensure validity and exclude artifactual events. mIPSCs with a rise time of more than 1ms were excluded to prevent the inclusion of events originating from a distal source. At least 50 events were sought for each recording, and the average peak amplitude, rise time (10–90%), charge transfer (area under the curve) and T _70_ (time required to decay by 70%), were analysed. The mIPSCs were digitally averaged by alignment at the midpoint of the rising phase. The mIPSC decay was fitted by monexponential ( *y(t) = Ae ^-t/τ^*) and biexponential (y(t) = A _fast_e ^(-t/τfast)^+ A _slow_e ^(-t/τslow)^) functions to determine which one was more appropriate ( *A* is amplitude, *t* is time and τ is the decay time constant). The standard deviations of the residuals of the monexponential and biexponential functions were measured and an *F* test applied. For the vast majority of mIPSCs analysed, the decay was best described by a biexponential function. Consequently, a mean weighted decay constant (τ _w_) was calculated to determine the relative contribution of each decay component. τ _w_ is a mathematical constant generated by considering the fast initial component of biexponential decay τ _1_ and the later slower component τ _2_. The value of τ _w_ is determined for the mean mIPSC for each cell by determining the relative proportion that τ _1_ and τ _2_ contribute to the biexponential decay. The following equation was used: τ _w_ = τ _fast_P _1_ + τ _slow_P _2_, where P _1_ and P _2_ represent the proportions of the synaptic current decay curve described by each component.

### Drug and solution preparation and administration

Salts used in the preparation of aECS and aCSF solutions were purchased from VWR (West Chester, Pennsylvania, USA). Strychnine (Sigma Chemicals, St. Louis, MO, USA), tetrodotoxin (Tocris, Bristol, UK) and bicuculline (Axxora, Nottingham, UK) and THIP (generous gift from B Ebert) were prepared as concentrated stock solutions in double-filtered water to be added to the aECS. Other compounds such as progesterone, ganaxolone, allopregnanolone, dihydroxy-progesterone, provera, indometacin, finasteride were purchased from Tocris or Sigma and prepared as concentrated stock solutions in dimethyl sulfoxide (DMSO). The cyclodextrins (CDs; Sigma) were dissolved directly into the aECS, or the intracellular solution. Brain and spinal cord slices were placed under the microscope into a transparent plastic chamber filled with aECS. The aECS was perfused through the chamber using a rate-adjustable gravity-based system consisting of hard plastic tubing that connected an oxygenated reservoir to the chamber. Simultaneously, a peristaltic pump system (Minipuls 3, Gilson, UK) drained the aECS from the opposite side of the chamber and recycled it back to the reservoir. The fluid-filled circuit also ran through a custom made heating system (G23, UCL, London, UK) controlled by a temperature monitoring system (School of Pharmacology, London, UK) that maintained the near physiological temperature of 35°C throughout.

### Behavioural experiments

***Drug administration.*** The drug, or vehicle, was administered by intra-peritoneal injection using a fine 1ml syringe and experiments were made pre- and post- injection in a randomised and blinded manner. Neurosteroids are lipophilic and have very limited solubility in aqueous solution, but can be solubilised in 0.9% saline using 2-hydroxy propyl β-cyclodextrin (β-CD) to facilitate administration ( Besheer *et al.*, 2010; Carter *et al.*, 1997; Reddy & Rogawski, 2010). Neurosteroids were therefore administered in 40% β-CD solution.

***Rotarod test.*** The rotarod test comprises an elevated rotating cylinder upon which a rodent is placed ( Jones & Roberts, 1968; Pritchett & Mulder, 2003). In order to avoid falling off the rotarod, the mouse must maintain constant motion; hence it is a test of forced motor activity ( Jones & Roberts, 1968; Pritchett & Mulder, 2003). To remain on the rotarod while it accelerates at a set rate the mouse requires balance and coordination. Mice were placed on the rotarod and the accelerating rotarod protocol was used. Specifically, the rod starts to rotate at 6 revolutions per minute (rpm) and is then increased in 4 rpm increments up to a maximum of 50 rpm. The experiment continues until the mouse falls off, or until the cut-off time of 300 seconds has elapsed.

***Thermal nociception.*** A modified tail flick test ( D’Armour & Smith, 1941; Mogil, 2009) was employed as follows: Thermal nociceptive thresholds were assessed by the immersion of 2cm of the animal’s tail in a water bath maintained at a specified temperature such as 40–50°C until the tail flick manoeuvre was initiated. The tail flick latency time was recorded and this parameter was used to compare the effect of neurosteroids *versus* injection of control vehicle and baseline measurements. A cut off time of 15 seconds was used to minimise the likelihood of tissue damage. The results of each experiment may be expressed in seconds, or as a percentage of the maximum possible effect (MPE) *i.e*. a percentage of 15 seconds.

***Mechanical nociception.*** A series of calibrated von Frey filaments (Ugo Basile, It) were employed to characterise mechanical nociceptive thresholds in WT and *ob/ob* mice. The mice were placed into clear plastic cubicles on top of a raised platform (Ugo Basile, Italy) with a meshed surface and allowed to acclimatise to the new environment for 30 minutes. The tip of the von Frey filament was pressed carefully onto the middle of the ventral (‘palmar’) surface of the hindpaw. Sufficient force to induce bending of the shaft of the filament was applied for up to 5 seconds and the presence of a withdrawal response noted if it occurred. The procedure was repeated until each hindpaw had received five presses of the filament. Only robust and immediate withdrawal responses were considered as positive. Each mouse could have a maximum score of 10 (five for each hindpaw). Testing would commence with the thinnest filament used and then progress to thicker filaments. Pilot studies were carried out to determine the optimal four filaments to be used in the mice: 0.16g, 0.4g, 0.6g and 1g filaments. They elicited a response in approximately 20%, 40%, 60% and 90% of occasions when applied to adult WT mice. This meant that these filaments could be used to test for the presence of mechanical hypersensitivty and nociception in the *ob/ob* mouse. This method was adapted from work published in rats with neuropathic sensitisation ( Meyer *et al.*, 2011).

***Statistical analysis.*** All data in the results section are expressed as the arithmetic mean ± the standard deviation (SD). The following statistical tests were employed where appropriate for the electrophysiological data: Student’s t test (Excel, Microsoft Office), One-way ANOVA, One-way and Two-way RM ANOVA (Sigmastat). The following non-parametric statistical tests were employed for the behavioural data: Mann-Whitney Rank Sum test, Kruskal Wallis one-way ANOVA on ranks and the Wilcoxon signed rank test (before & after; Sigmastat).

## Results

### The decay time of GABA _A_R mIPSCs decreases with development at three levels of the pain pathway

Electrophysiological recordings were made from C57/Bl6 mice in lamina II (LII) of the dorsal horn of the spinal cord, the nucleus reticularis (nRT) of the thalamus and layer 2/3 of the cortex (pyramidal neurons). These neurons were selected due to their modulatory role in nociceptive transmission, associated with the perception of pain in humans ( Clasca *et al.*, 2012; Cox *et al.*, 1997; DeFelipe & Farinas, 1992; Gentet & Ulrich, 2003). Previous work ( Brown *et al.*, 2015) on murine neurons of the cortex and thalamocortical neurons of the ventrobasal (VB) thalamus revealed that fluctuations in the endogenous neurosteroid tone during development (P7–P24) influenced the duration of miniature inhibitory postsynaptic currents (mIPSCs).

**Figure 2.**
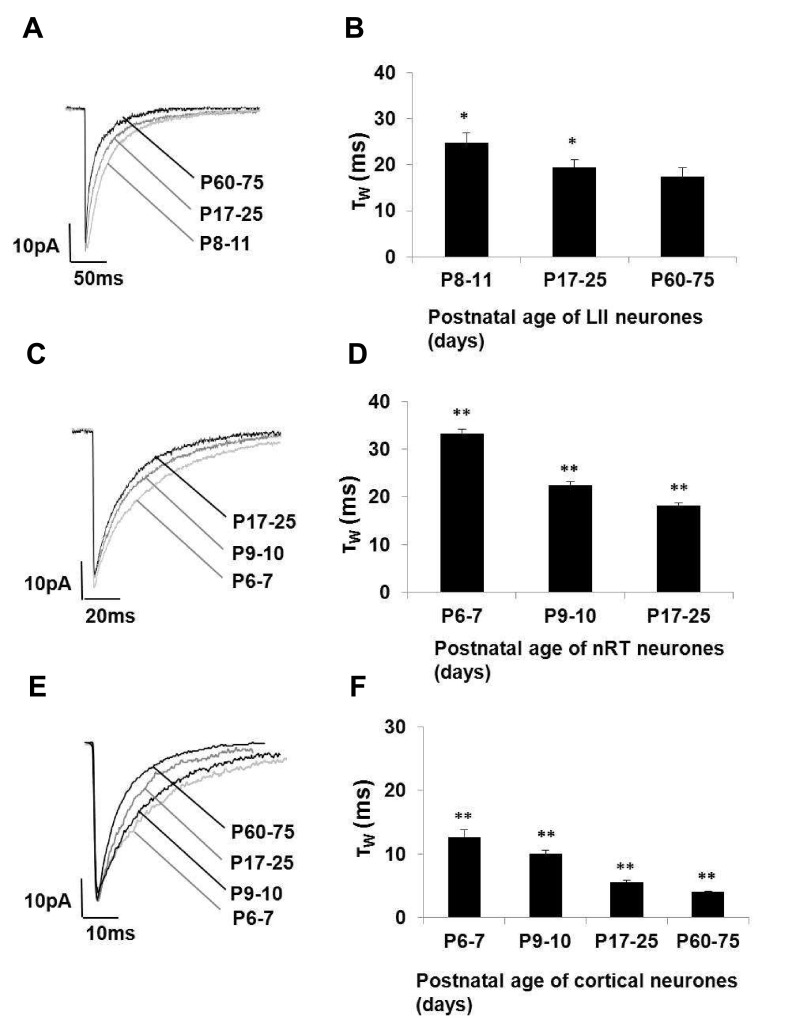
The decay time of GABA _A_R mIPSCs of neurons from three levels of the pain pathway decreases with development. ( **A**) Superimposed exemplar GABA _A_R-mediated mIPSCs recorded from representative spinal neurons from three stages of development: P8–11 (light grey), P17–25 (grey) and P60–75 (black). Note the reduction of GABA _A_R mIPSC decay time that occurs with development. ( **B**) Histogram illustrating the shortening of GABA _A_R mIPSCs with development (One-way ANOVA * *P* < 0.05; n = 13–31). ( **C** & **D**) A parallel developmental change is observed in nRT neurons One-way ANOVA *P* < 0.05. *Post hoc* Newman Keul’s test revealed significant differences between all groups, ** *P* < 0.05; n = 14–24). ( **E** & **F**) A parallel developmental change is observed in L2/3 cortical neurons One-way ANOVA *P* < 0.05. *Post hoc* Newman Keul’s test revealed significant differences between all groups, ** *P* < 0.05; n = 7–35).

Previous studies of LII neurons revealed the mIPSC time course of decay may reduce with development, a perturbation that may be caused by the loss of an endogenous steroid tone ( Keller *et al.*, 2001; Keller *et al.*, 2004; Maguire *et al.*, 2014; Rajalu *et al.*, 2009). mIPSC decay (τ _W_) decreased with development (P8–11 = 24.8 ± 2 ms; n = 26; P17–25 = 19.4 ± 1.8 ms, n = 31; P60–75 = 17.5 ± 1.8 ms, n = 13; One-way ANOVA, *P* <0.05; Figure 2A,B). The τ _W_ is approximately equivalent to the time taken for the mIPSC to decrease by 67% from the peak amplitude. The unfavourable signal-to-noise of individual mIPSCs precludes the accurate fitting of the τ _W_ to individual mIPSCs. Therefore, this function is fitted to the mean mIPSC derived for each neuron.

The nRT is the main source of GABAergic input into the thalamus and there are reciprocal GABAergic loops of innervation between nRT neurons and those of the VB. The two types of neurons, nRT and VB; regulate each other’s function by this mutual inhibitory mechanism ( Arcelli *et al.*, 1997; Cox *et al.*, 1997; Gentet & Ulrich, 2003; Guillery & Harting, 2003). The inter-relationship between the nRT and VB acts to modulate nociceptive transmission ( Huh *et al.*, 2012). Recordings from nRT neurons were made at three developmental stages: P6-7, P9-10 and P17-25. It is not practical to make such recordings from mice over the age of P25 due to the high density of axonal projections ( Cox *et al.*, 1997; Pinault & Deschenes, 1998). The mIPSC decay (τ _W_) of nRT neurons decreased significantly with development (P6-7 = 33.2 ± 1 ms, n = 24; P9-10 = 22.5 ± 0.7 ms, n = 14; P17-25 = 18.2 ± 0.6 ms, n = 32; One-way ANOVA, *P* < 0.05; *post hoc* Newman Keul’s test revealed significant differences between all groups, *P* <0.05; Figure 2C,D).

Further experiments revealed that GABA _A_Rs from neurons at all three levels of the pain pathway are sensitive to modulation by neurosteroids (see Additional material). The endogenous neurosteroid tone was explored using γ-cyclodextrin (γ-CD), a barrel-shaped molecule known to sequester neurosteroids ( Shu *et al.*, 2004; Shu *et al.*, 2007). The three principle types of CD are the α-CD, β-CD and γ-CD, they have internal diameters of 5.2 nm, 6.4 nm and 8.3 nm respectively ( Cooper *et al.*, 2005; Davis & Brewster, 2004). The largest of these, γ-CD is the most effective for the sequestration of neurosteroids ( Brown *et al.*, 2015; Shu *et al.*, 2004; Shu *et al.*, 2007). The mIPSC decay (τ _W_) in WT mice was significantly decreased in the presence of γ-CD, but not by α-CD, or β-CD (see Additional material). γ-CD has been reported to have no direct effect on the GABA _A_R ( Shu *et al.*, 2004; Shu *et al.*, 2007) and neither of the smaller molecules, α-CD or β-CD, had an impact on mIPSC τ _W_ ( Table S2). Intracellular application of γ-CD *via* the recording pipette was found to be the optimal method of application (see Additional material) and is consistent with the hypothesis that the GABA _A_R-active neurosteroids are synthesised within the pain pathway neurons themselves ( Akk *et al.*, 2005; Chisari *et al.*, 2009; Tsutsui, 2008).

### Pipette-applied γ-CD reduces decay time of GABA _A_R mIPSCs of layer 2/3 pyramidal cortical neurons at two stages of development

The mIPSC decay (τ _W_) of L2/3 cortical neurons at two stages of maturity (P9–10 and P60–75) was significantly decreased in the presence of intracellular γ-CD ( *P* <0.05, Table S1). These data are in contrast to the lack of effect of γ-CD observed in nRT neurons at P9–10 and P17–24. However, the data for P9/10 L2/3 cortical neurons are consistent with data published previously ( Brown *et al.*, 2015). Different regions of the nervous system reach maturation at different ages and it is possible that this may account for the regional variations observed. Interestingly, the endogenous neurosteroid tone that previously appeared to be lost during maturation re-emerges in the adult mouse cortex, which may have a significant physiological role.

### A comparison of synaptic GABA _A_R mIPSCs of cortical layer 2/3 pyramidal neurons in adult WT, *ob/ob* and *db/db* mice incorporating the use of cyclodextrin

As the behavioural studies were to be conducted in adult mice, it was decided to make recordings from adult layer 2/3 cortex neurons for three reasons: 1) Viable recordings of nRT neurons of older animals are compromised by the high density of axonal projections ( Cox *et al.*, 1997; Pinault & Deschenes, 1998). 2) Values for the mean τ _W_ of L2/3 cortical GABA _A_R mIPSCs are relatively homogenous, in contrast to the mean τ _W_ values of GABA _A_R mIPSCs of LII neurons which are heterogenous ( Mitchell *et al.*, 2007), which makes inter-group comparison more difficult. 3) Layer 2/3 pyramidal neurons of the somatosensory cortex are part of the pain pathway.

The developmental age of P60–75 was chosen because it facilitated a comparison with the *ob/ob* mouse model of type-2 diabetes mellitus (T2DM). The *ob/ob* mouse develops super-morbid obesity and exhibits a neuropathic phenotype and consequently develops hypersensitivity to pain by the age of P60–75 ( Drel *et al.*, 2006; Latham *et al.*, 2009). To date, there are no published reports of the electrophysiological characterisation of GABA _A_R function for the *ob/ob* mouse.

Mice were weighed in order to confirm the presence of obesity. The *ob/ob* and *db/db* mice (P60–75) both had significantly greater body weights than the respective WT animals of the same age ( Table S3, *P* < 0.05). These data are consistent with the literature ( Bates *et al.*, 2005; Latham *et al.*, 2009).

**Figure 3.**
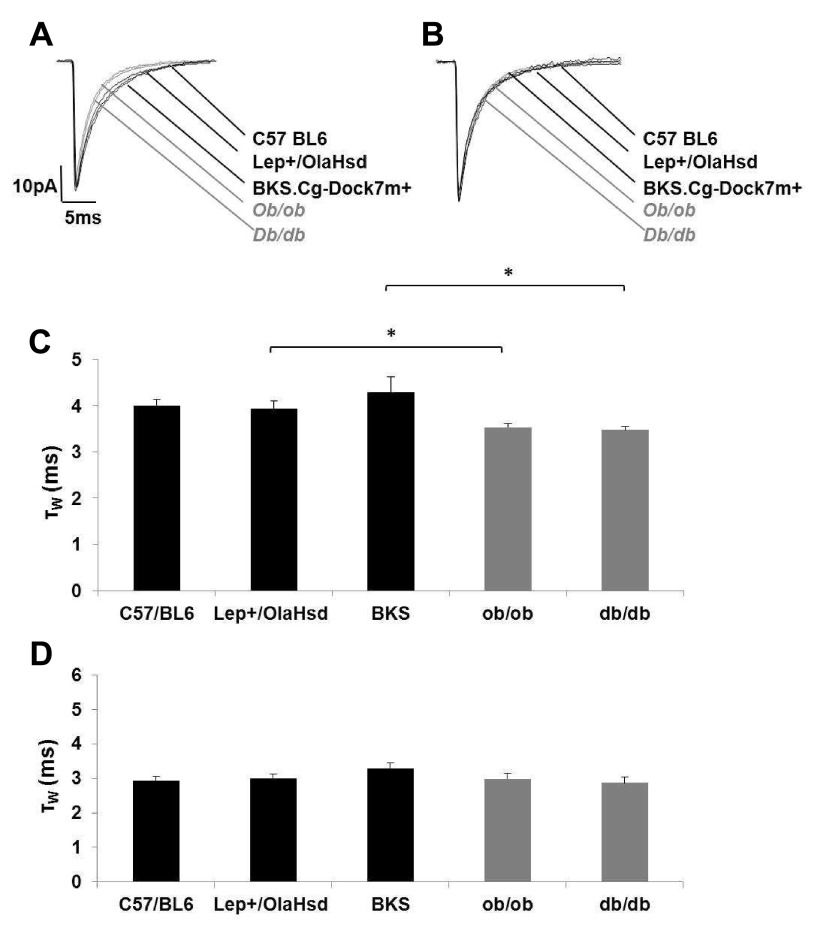
Mature cortical neurons of *ob/ob* & *db/db* mice exhibit shorter GABA _A_R mIPSCs compared to those from three different WT strains. Intracellular γ-CD reduces the duration of GABA _A_R mIPSCs of mature cortical neurons from different types of mice to a similar baseline level. ( **A**) Superimposed exemplar GABA _A_Rs mIPSCs from three WT strains (black) and two diabetic phenotypes (grey). ( **B**) Superimposed exemplar GABA _A_R-mediated mIPSCs from three WT strains (black) and two diabetic phenotypes (grey) with 0.5 μM γ-CD administered intracellularly. ( **C**) Histogram illustrating the shorter cortical GABA _A_R mIPSC τ _w_ of the diabetic mice and their corresponding WT strains (n = 8–25; Student’s unpaired t test * *P* <0.05). ( **D**) Histogram illustrating that there was no significant difference between all five strains of mice in the presence of intracellular (γ-CD n = 5–15; One-way ANOVA *P* > 0.05).

To exclude the presence of a direct effect of leptin itself recordings were additionally made in the *db/db*, which is able to synthesise leptin but lacks the receptor. There was a modest but significant reduction in the mIPSC decay (τ _W_) of cortical neurons between the diabetic mice and the corresponding WT littermates, but no significant difference in the τ _W_, between the three WT strains ( *P* <0.05; Table S4, Figure 3A,C).

Recordings were made to determine that neither the strain, nor the lack of leptin (directly) was an important factor in the shortening of mIPSC τ _W_ with γ-CD. In the presence of intracellular γ-CD, there was no significant difference in the mIPSC τ _W_ of between all five types of mice ( Table S5, Figure 3D, *P* > 0.05). These findings suggest that synaptic GABA _A_R function is very similar across all these strains of mice included in the study when the endogenous neurosteroid tone is removed by γ-CD. The results are also consistent with the hypothesis that there is a neurosteroid tone at P60–75, but that it is reduced in both mouse models of diabetic neuropathy. However, the data do not exclude the possibility that the sensitivity of L2/3 cortical GABA _A_Rs to neurosteroids may be reduced.

### A comparison of the effect of neurosteroids on cortical layer 2/3 neurons in adult WT, *ob/ob* and *db/db* mice

**Figure 4.**
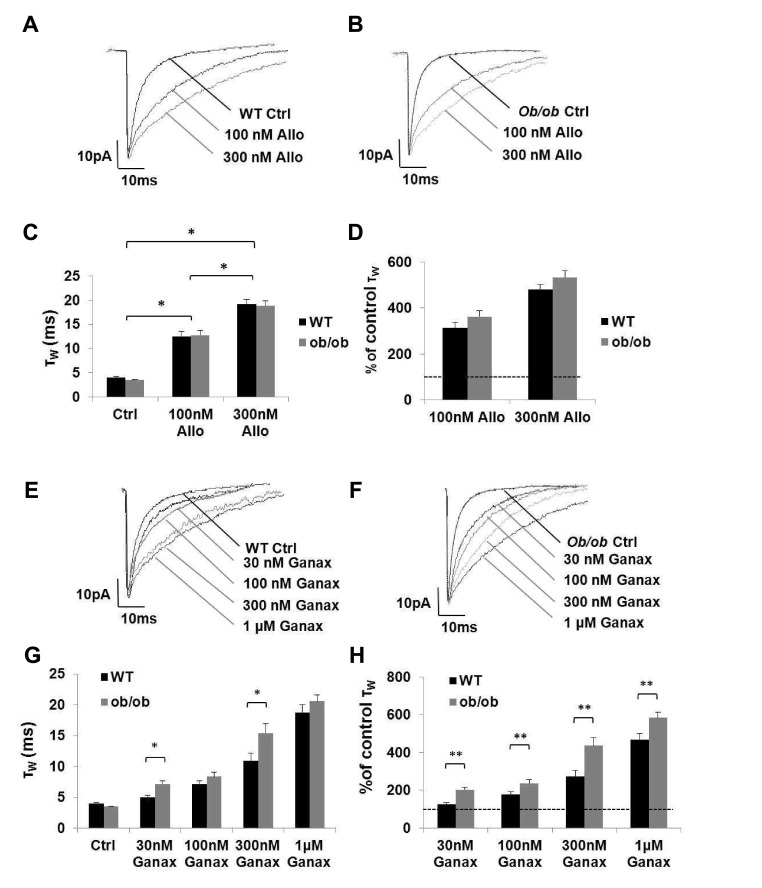
Prolonged exposure (~2 hrs) of WT & *ob/ob* mature cortical neurons to allopregnanolone (100–300 nM) greatly enhances the function of synaptic GABA _A_Rs by a similar margin while ganaxolone has an exaggerated effect in *ob/ob* mice. ( **A** & **B**) Superimposed exemplar GABA _A_R mIPSCs from representative control cortical neurons from mature WT and *ob/ob* mice respectively and from equivalent neurons after ~2 hour brain slice incubation with 100 nM and 300 nM allopregnanolone. ( **C**) Histogram illustrating the concentration-dependent effect of allopregnanolone on the cortical GABA _A_R mIPSCs of *ob/ob* mice (n = 6–25; One-way ANOVA *P* <0.05. *Post hoc* Newman Keuls test revealed significant differences between control and both concentrations of allopregnanolone, which increased τ _w_ to 362 ± 27% and 534 ± 28% of control respectively * *P* <0.05). Note that there is no significant difference in response between the two types of mice (n = 8–9; One-way RM ANOVA *P* > 0.05). ( **D**) Histogram comparing the concentration-dependent effects of allopregnanolone on the duration of GABA _A_Rs mIPSC τ _w_ expressed as a percentage of control for WT (black) and *ob/ob* (grey) mice. The histogram illustrates that there is no significant difference in the effect of 300 nM allopregnanolone on the cortical GABA _A_Rs mIPSCs of WT and *ob/ob* neurons (n = 8–9; One-way RM ANOVA *P* > 0.05). ( **E** & **F**) Superimposed exemplar GABA _A_R mIPSCs from representative control cortical neurons from mature WT and *ob/ob* mice respectively and from equivalent neurons after ~2 hour brain slice incubation with 30 nM - 1 μM ganaxolone. ( **G**) Histogram illustrating the concentration-dependent effect of ganaxolone 30 nM - 1 μM on the GABA _A_Rs mIPSCs of *ob/ob* mice (grey bars; One-way ANOVA *P* <0.05). Note the exaggerated effect of ganaxolone incubation treatment on the cortical GABA _A_Rs mIPSCs of *ob/ob* mice (grey bars) in comparison to WT mice (black bars) for 30 nM and 300 nM ganaxolone (One-way RM ANOVA, *P < 0.05 respectively). ( **H**) Histogram comparing the concentration-dependent effects of ganaxolone on the duration of GABA _A_Rs mIPSC τ _w_ expressed as a percentage of control for WT (black) and *ob/ob* (grey) mice. The histogram illustrates that ganaxolone has an exaggerated effect in *ob/ob* mice One-way RM ANOVA **P < 0.05). Ctrl = control; Allo = allopregnanolone; Ganax = ganaxolone.

The lipophilic intravenous anaesthetics etomidate and propofol, which in common with neuroactive steroids enhance the function of GABA _A_Rs, require relatively prolonged incubation times to approach equilibrium in a brain slice preparation- over 1–2 hours ( Benkwitz *et al.*, 2007; Gredell *et al.*, 2004). It is conceivable that the same is true for neurosteroids ( Li *et al.*, 2007) therefore recordings were made after incubation treatment with allopregnanolone and ganaxolone. In contrast to the relatively modest prolongation of mIPSCs described above with an acute steroid application protocol (τ _W_: control = 4.0 ± 0.3 ms, n = 7; allopregnanolone 1 μM = 4.5 ± 0.4 ms, n = 7; paired Student’s t test, P <0.05), a 2-hour incubation of the brain slice preparation with nanomolar concentrations of allopregnanolone (100 – 300 nM) produced a dramatic concentration-dependent increase of the WT GABA _A_R mIPSCs ( Table S6, Figure 4A,C,D; *P* <0.05). These results indicate that allopregnanolone is a potent modulator of synaptic GABA _A_Rs in mature cortical neurons, but additionally demonstrate that the steroid effect is greatly underestimated when applied acutely. The large difference between acute bath application and the 2-hour incubation (see Additional material) is probably a consequence of the time required for the steroid to approach equilibrium within the brain slice. No such effect is observed in time-matched controls.

**Figure S1.**
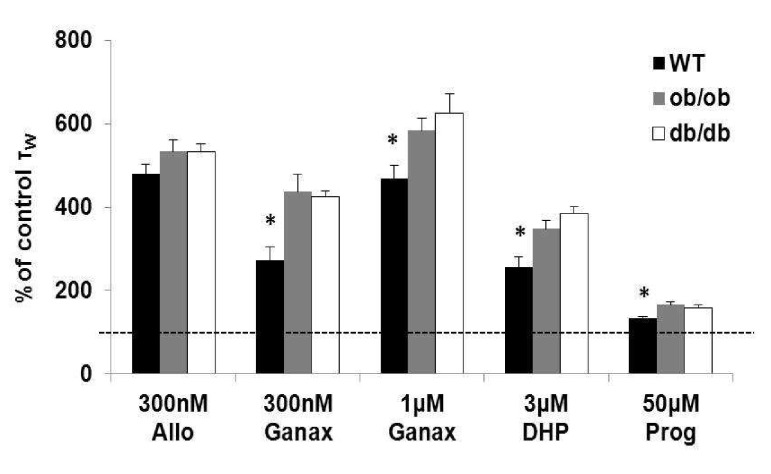
Relative differences in the potencies of certain neurosteroidal compounds. Histogram summarising the relative differences in the potencies of certain neurosteroidal compounds (allopregnanolone, ganaxolone, DHP and progesterone) in three strains of mice (WT, *ob/ob* and *db/db*) after ~2 hours brain-slice exposure. When the mIPSC τ _w_ were normalised for comparison, allopregnanolone and ganaxolone, which modulate the GABA _A_R directly, have the greatest potency, while the neurosteroid precursors DHP and progesterone have the least potency. There is no significant difference in the effect of allopregnanolone on the three strains of mice (n = 6–9, *P* > 0.05). In contrast, the precursor DHP had a greater impact on the diabetic mice n = 8–10; One-way RM ANOVA * *P* < 0.05. *Post hoc* Newman Keul’s test revealed significant differences between the WT and both types of diabetic mice, * *P* < 0.05, but there was no intergroup difference between the *ob/ob* and *db/db* mice, *P* > 0.05). The precursor progesterone, also had a greater impact on the diabetic mice, n = 6–12; One-way RM ANOVA *P* < 0.05. *Post hoc* Newman Keul’s test revealed significant differences between the WT and both types of diabetic mice, *P* < 0.05, but there was no intergroup difference between the *ob/ob* and *db/db* mice, *P* > 0.05). Ctrl = control; Allo = allopregnanolone; Ganax = ganaxolone; DHP = dihydroxyprogesterone; Prog = progesterone.

A 2-hour incubation of the *ob/ob* brain slice preparation with allopregnanolone produced a clear concentration-dependent prolongation of GABA _A_R mIPSC τ _W_ ( Table S6, Figure 4B–D; *P* <0.05). The effect of allopregnanolone was similar for WT, *ob/ob* and *db/db* mice ( Table S6; *P* > 0.05). When the data were normalised to reflect the control mIPSC τ _W_, there was no significant difference in the effect of between the three types of mice ( Figure S1, *P* > 0.05). These results suggest that the sensitivity of cortical GABA _A_Rs to allopregnanolone in diabetic mice is similar to age-matched WT.

A 2-hour incubation with ganaxolone produced a significant concentration-dependent increase of the WT mIPSC τ _W_, although the magnitude of the effect was less than that induced by allopregnanolone ( Table S7, Figure 4E,G,H; *P* <0.05). In the *ob/ob* and *db/db* brain slices incubated with ganaxolone the mIPSC τ _W_ prolongation was comparatively greater than for equivalent WT neurons, but there was no significant difference between the *ob/ob* and *db/db* ( Table S7; *P* > 0.05). These observations contrast with that of allopregnanolone. These findings suggest that there is a difference in the effect of ganaxolone incubation treatment (but not allopregnanolone) in the *ob/ob* and *db/db* mice compared to the WT mice.

### Can mature layer 2/3 cortical neurons from WT, *ob/ob* and *db/db* mice synthesise neurosteroids?

Progesterone and its metabolite dihydroxy-progesterone (DHP) do not modulate GABA _A_Rs directly ( Belelli & Herd, 2003; Brown *et al.*, 2015), but require the activity of the enzymes 5α-R and 3α-HSD in order to synthesise allopregnanolone ( Figure 1; Schumacher *et al.*, 2012; Stoffel-Wagner, 2003).

**Figure 5.**
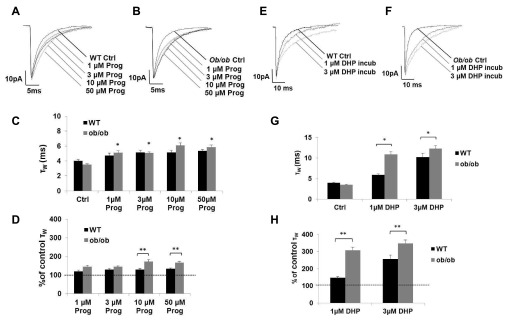
Prolonged exposure (~2 hrs) of mature cortical neurons to progesterone (1–50 μM) and DHP (1–3 μM) enhances the function of synaptic GABA _A_Rs, suggesting that both WT & *ob/ob* neurons can synthesise allopregnanolone. ( **A** & **B**) Superimposed exemplar GABA _A_R mIPSCs from a representative control mature WT and *ob/ob* cortical neurons and from equivalent neurons after ~2 hour brain slice incubation with 1 μM – 50 μM progesterone. ( **C**) Histogram illustrating the significant but modest effect of progesterone incubation treatment on WT and *ob/ob* cortical GABA _A_Rs mIPSCs (grey) that was not concentration-dependent (One-way ANOVA * *P* <0.05). ( **D**) Histogram illustrating the concentration-independent effect of progesterone on the duration of GABA _A_Rs mIPSC τ _w_ expressed as a percentage of control. Note that when the effect of progesterone 50 μM was expressed as a percentage of the representative control, the steroid had a greater impact on the *ob/ob* mice (n = 7–12, One-way RM ANOVA ** *P* < 0.05). ( **E** & **F**) Superimposed exemplar GABA _A_R mIPSCs from a representative control mature WT and *ob/ob* cortical neurons and from equivalent neurons after ~2 hour brain slice incubation with 1 – 3 μM DHP. ( **G**) Histogram illustrating the significant concentration-dependent effect of DHP on the duration of WT and *ob/ob* cortical GABA _A_Rs mIPSCs (n = 10–25; One-way ANOVA *P* <0.05). Note the exaggerated response of the *ob/ob vs.* the WT cortical neurons for 1–3 μM DHP (n = 9–10, one-way ANOVA * *P* < 0.05). ( **H**) Histogram illustrating that 1 – 3 μM DHP has an exaggerated effect in *ob/ob* (grey) *vs.* WT (black) mice when the effect is expressed as a percentage of the respective control value (One-way RM ANOVA ** *P* < 0.05). Ctrl = control; DHP = dihydroxyprogesterone; Prog = progesterone.

A 2-hour incubation with progesterone produced a relatively modest prolongation of mIPSC τ _W_ in WT mice. The highest concentration of progesterone tested was only slightly more effective than the lowest concentration investigated here ( Table S8, Figure 5A,C; *P* <0.05). These results suggest that the enzymatic function (5α-R and 3α-HSD) is intact and neurosteroids may be synthesised with brain slice incubation of the precursor.

Progesterone incubation treatment also produced a similarly modest prolongation of mIPSC τ _W_ in the *ob/ob* and *db/db* ( Table S8, Figure 5B,C; *P* <0.05). When the data for τ _W_ were normalised. When the effects of 50 μM progesterone were expressed as a percentage of the representative control, the steroid had a greater impact on the diabetic mice ( *P* < 0.05), but there was no intergroup difference between the *ob/ob* and *db/db* mice ( *P* > 0.05; Table S8, Figure S1; Figure 5D).

Bath application of DHP (3μM) had no effect on the GABA _A_R mIPSCs in WT neurons (τ _W_: Control = 5 ± 0.3 ms, n = 4; 3 μM DHP = 4.7 ± 0.3 ms, n = 4, *P* > 0.05). These findings are consistent with previous work on VB neurons ( Brown *et al.*, 2015). The lack of effect after the acute application of DHP contrasts to the modest effects of acutely applied allopregnanolone and ganaxolone (see Additional material). Contrastingly, 2 hours of incubation with DHP (1 – 3 µM) produced a significant, concentration-dependent prolongation of GABA _A_R mIPSCs in WT (P <0.05; Table S9, Figure 5E,G). These results indicate that mature WT L2/3 cortical neurons have intact 3α-HSD enzymatic function and are able to convert DHP into the active metabolite allopregnanolone. 3μM DHP incubation produced a more pronounced effect in the *ob/ob* and *db/db* mice compared to the WT ( *P* <0.05, Table S9), indicating that not only is 3α-HSD enzymatic function preserved in mature mice with T2DM, but may be up-regulated.

### Selective inhibition of enzymes within the neurosteroid synthesis pathway in mature layer 2/3 cortical neurons from WT, *ob/ob* and *db/db* mice

Finasteride itself has no direct effect on GABA _A_R mIPSCs, but pre-treatment with this 5α-R enzyme inhibitor prevents the conversion of progesterone into GABA _A_R-active neurosteroid ( Sanna *et al.*, 2004). Recordings were made after at least 2 hours of incubation with finasteride and progesterone. Finasteride alone had no effect on WT mIPSC τ _W_ but it did prevent the effect of progesterone ( Table S10; *P* <0.05). Finasteride alone also had no effect on *ob/ob* mIPSC τ _W_ of mice, but it did prevent the effect of progesterone ( Table S10; *P* < 0.05) indicating that progesterone requires the 5α-R for it to be converted to its’ active metabolites, (although do not prove neurosteroid synthesis by the neurons itself). Finasteride alone had no effect on mIPSC τ _W_, despite the suggested presence of a modest endogenous neurosteroid tone in mature WT mice. This apparent paradox may be explained by comparing how finasteride and γ-CD act. γ-CD will remove the endogenous neurosteroid present, whereas although finasteride should prevent new allopregnanolone synthesis, it will have little impact on that already present. Therefore, the apparent lack of an effect of finasteride may reflect the relatively slow turnover of pre-synthesised allopregnanolone and is consistent with the literature ( Brown *et al.*, 2015).

### The 3α-HSD enzyme inhibitor provera suppresses the effects of DHP, but not ganaxolone on GABA _A_R-mediated mIPSCs of mature cortical neurons

**Figure S2.**
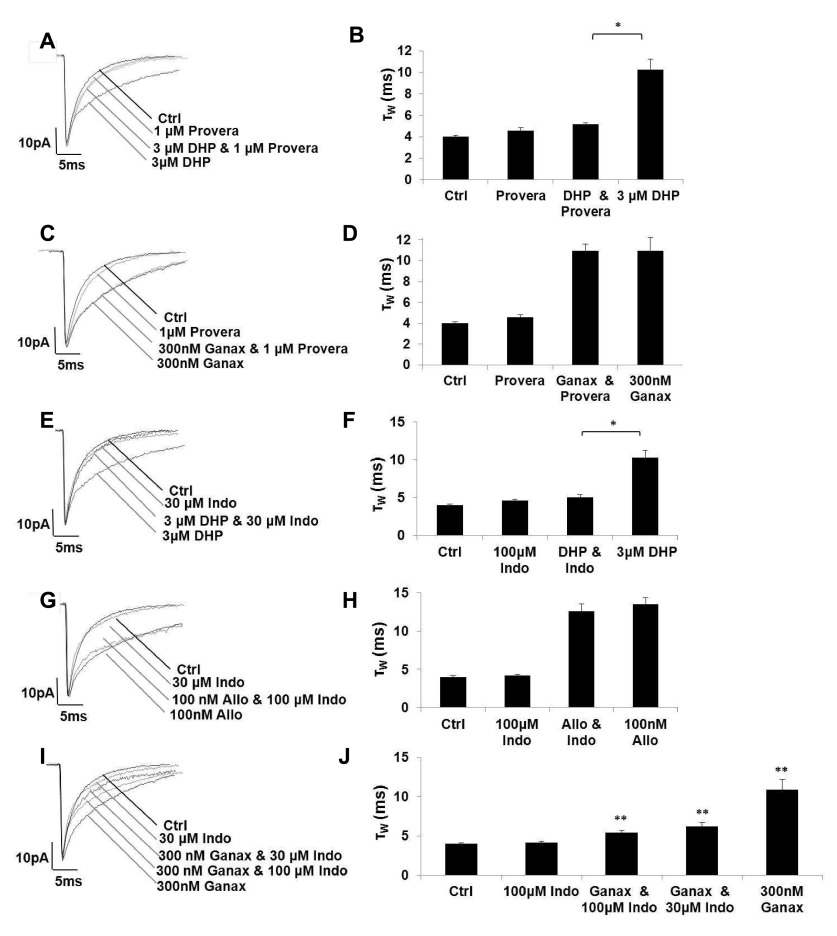
The 3α-HSD enzyme inhibitor provera suppresses the effects of DHP, but not ganaxolone on GABA _A_R-mediated mIPSCs of mature cortical neurons. The 3α-HSD enzyme inhibitor indometacin suppresses the effects of DHP and ganaxolone, but not those of allopregnanolone on GABA _A_R-mediated mIPSCs of mature cortical neurons. ( **A**) Superimposed exemplar averaged GABA _A_R mIPSCs from a representative control cortical neuron and from equivalent neurons after ~2 hour pre-incubation of the brain slice with provera (1 μM), DHP (3 μM), or both. To facilitate comparison of their kinetics, the amplitude of the mIPSCs are normalised to that of the control averaged mIPSC ( **B**) Histogram illustrating that provera prevents the effect of DHP pre-incubation to prolong GABA _A_R-mediated mIPSCs (τ _w_ in ms; one-way ANOVA *P* <0.05. *Post hoc* Newman Keul’s test revealed differences for DHP (3 μM) with or without provera (1 μM), * *P* <0.05, n = 7–9). ( **C**) Superimposed exemplar averaged GABA _A_R mIPSCs from a representative control cortical neuron and from equivalent neurons after ~2 hour pre-incubation of the brain slice with provera (1 μM), ganaxolone (300 nM), or both. ( **D**) Histogram illustrating that provera did not prevent the effect of ganaxolone on the duration of GABA _A_R mIPSCs (τ _w_ in ms; one-way ANOVA *P* <0.05. *Post hoc* Newman Keul’s test revealed no difference for the effects of ganaxolone (300 nM) with, or without provera (1 μM), *P* >0.05, n = 7–10). ( **E**) Superimposed exemplar averaged GABA _A_R mIPSCs acquired from a representative control cortical neuron and from equivalent neurons after ~2 hours pre-incubation of the brain slice with indometacin (30 μM), DHP (3 μM), or both. ( **F**) Histogram illustrating that indometacin prevents the effect of DHP on the duration of the GABA _A_R mIPSCs (τ _w_ in ms, one-way ANOVA *P* <0.05. *Post hoc* Newman Keul’s test revealed differences for DHP 3 μM with or without indometacin 100 μM, * *P* <0.05, n = 6–9). ( **G**) Superimposed exemplar averaged GABA _A_R mIPSCs acquired from a representative control neuron and from equivalent neurons after an ~2 hour pre-incubation of the brain slice with indometacin (100 μM), allopregnanolone (100 nM), or both. ( **H**) Histogram illustrating that indometacin does not prevent the effect of allopregnanolone to prolong the duration of the GABA _A_R-mediated mIPSC (τ _w_ in ms, one-way ANOVA *P* >0.05. *Post hoc* Newman Keul’s test revealed no differences for allopregnanolone with or without indometacin, *P* >0.05, n =7–9). ( **I**) Superimposed exemplar averaged GABA _A_R mIPSCs acquired from a representative control neuron and from equivalent neurons after ~2 hour incubation of the brain slice with indometacin (100 µM), ganaxolone (300 nM), or both. ( **J**) Histogram illustrating that indometacin in a concentration-dependant manner (30 – 100 µM) prevents the effect of ganaxolone to prolong the GABA _A_R-mediated mIPSC (τ _w_ in ms; one-way ANOVA *P* < 0.05. *Post hoc* Newman Keul’s test revealed differences for ganaxolone 300 nM with or without indometacin 30 – 100 μM, ** *P* < 0.05, n = 8–10. Ctrl = control; Allo = allopregnanolone; Ganax = ganaxolone; DHP = dihydroxyprogesterone; Indo = indometacin.

Recordings were made in WT mice after at least 2 hours of incubation with provera and DHP, or provera and ganaxolone. Provera alone exerted a modest effect on mIPSC τ _W_ but, in addition, it prevented the effect of DHP ( Table S11, Figure S2 A,B; *P* <0.05). Provera did not inhibit the effect of ganaxolone on τ _W_ in WT mice ( Table S11, Figure S2 C,D, *P* >0.05). These results confirm that DHP requires enzymatic conversion by 3α-HSD for it to induce a prolongation of τ _W_. In contrast, ganaxolone was unaffected by 3α-HSD inhibition with provera.

### The 3α-HSD enzyme inhibitor indometacin suppresses the effects of DHP and ganaxolone, but not those of allopregnanolone on GABA _A_R-mediated mIPSCs of mature cortical neurons

Indometacin alone had no effect on τ _W_, but it also prevented the effect of DHP ( Table S12, Figure S2 E,F; *P* <0.05). By contrast, indometacin had no effect on the prolongation of mIPSC τ _W_ by pre-incubated allopregnanolone ( Table S13, Figure S2 G,H; *P* >0.05) confirming that allopregnanolone does not require 3α-HSD in order to modulate GABA _A_R mIPSCs. Indometacin did not increase the effect of allopregnanolone by potentially inhibiting its metabolism to an inactive form. These experiments with another 3α-HSD enzyme inhibitor confirm that DHP requires conversion to a more active form by 3α-HSD in order for it to induce prolongation of GABA _A_R mIPSC decay time. By contrast, indometacin exhibited concentration-dependent inhibition of ganaxolone incubation treatment on τ _W_ ( Table S13, Figure S2 I,J, *P* <0.05). These results with ganaxolone were unexpected given the lack of impact that indometacin had on the effectiveness of allopregnanolone. Indeed, inhibiting 3α-HSD with provera had no impact on ganaxolone, but prevented the effect of DHP on GABA _A_R mIPSC τ _W_. This raises the question as to whether ganaxolone is an allosteric modulator of the GABA _A_R and a precursor to a more active neurosteroid such as allopregnanolone. Alternatively, it raises the question of whether indometacin could be a silent competitive steroid antagonist at the GABA _A_R and prevent the action of ganaxolone by that mechanism (explored in next section).

### The effect of pipette-applied neurosteroids on layer 2/3 cortical GABA _A_R mIPSCs in mature WT mice (P60-75)

**Figure S3.**
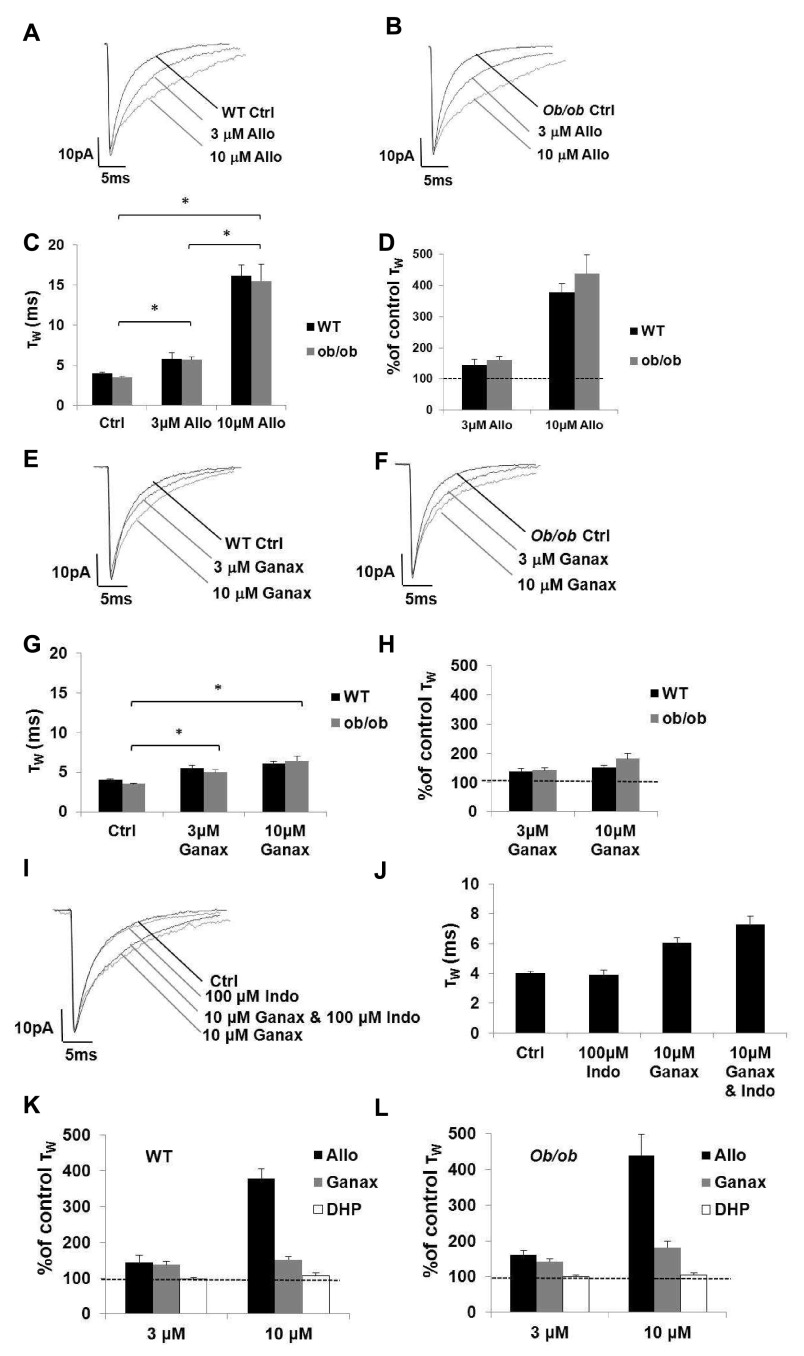
The intracellular application of allopregnanolone or ganaxolone to mature cortical neurons enhances synaptic GABA _A_R function by a similar margin in WT and *ob/ob* mice. The 3α-HSD enzyme inhibitor indometacin does not suppress the acute effects of intracellularly applied ganaxolone on GABA _A_R mIPSCs of mature cortical neurons (in contrast to its inhibitory effect on ganaxolone incubation treatment). ( **A** & **B**) Superimposed exemplar GABA _A_R mIPSCs from a representative control WT and *ob/ob* cortical neurons and from equivalent neurons with 3 – 10 μM allopregnanolone administered intracellularly. ( **C**) Histogram illustrating the concentration-dependent effect of the intracellular application of allopregnanolone on the duration of GABA _A_Rs mIPSC τ _w_ in WT and *ob/ob* cortical neurons (n = 5–25; One-way ANOVA * *P* < 0.05). ( **D**) Histogram illustrating the concentration-dependent effect of allopregnanolone on the duration of GABA _A_Rs mIPSC τ _w_ of WT (black) and *ob/ob* cortical neurons (grey) expressed as a percentage of control. There was no significant difference in the effect of allopregnanolone on WT *vs. ob/ob* cortical GABA _A_Rs mIPSCs (n = 5–7; One-way RM ANOVA *P* > 0.05). ( **E** & **F**) Superimposed exemplar GABA _A_Rs mIPSCs from a representative control WT and *ob/ob* cortical neurons and from equivalent neurons with 3–10 μM ganaxolone administered intracellularly. ( **G**) Histogram illustrating the concentration-dependent effect of the intracellular application of ganaxolone on the duration of GABA _A_Rs mIPSC τ _w_ in WT and *ob/ob* cortical neurons (n = 6–25; One-way ANOVA * *P* < 0.05). ( **H**) Histogram illustrating the concentration-dependent effect of ganaxolone on the duration of GABA _A_Rs mIPSC τ _w_ of WT (black) and *ob/ob* cortical neurons (grey) expressed as a percentage of control. There was no significant difference in the effect of ganaxolone on WT *vs. ob/ob* cortical GABA _A_Rs mIPSCs (n = 6; One-way RM ANOVA *P* > 0.05). ( **I**) Superimposed exemplar averaged GABA _A_R-mediated mIPSCs acquired from a representative control mature L2/3 cortical neuron and from equivalent neurons after the intracellular application of ganaxolone (10 μM), indometacin (100 μM) or both. ( **J**) Histogram illustrating that intracellular indometacin made no impact on the effectiveness of intracellular ganaxolone (Student’s unpaired t tests *P* > 0.05 for ganaxolone 10 μM *vs*. ganaxolone 10 μM with indometacin 100 μM, n = 4–6). ( **K** & **L**) Histograms illustrating the lack of effect of the intracellular application of 3–10 μM DHP (white bars) on the duration of WT and *ob/ob* cortical GABA _A_Rs mIPSC τ _w_ (One-way ANOVA *P* > 0.05) in contrast to the effectiveness of allopregnanolone and ganaxolone. Ctrl = control; Allo = allopregnanolone; Ganax = ganaxolone; DHP = dihydroxyprogesterone.

Acute allopregnanolone had only a modest effect on the GABA _A_R-mediated mIPSCs decay time (see Additional material), but was far more efficacious in this respect when brain tissue slices were incubated with steroid for > 2 hours, suggesting that the steroid is relatively slow to equilibrate within the tissue. It has been proposed that endogenous neurosteroids may be synthesised in the postsynaptic neuron and act in an autocrine manner to influence GABA-ergic transmission ( Agis-Balboa *et al.*, 2006; Lambert *et al.*, 2009). It is implicit in this model that intracellular steroid would modulate the GABA _A_Rs of the postsynaptic neuron and the recording pipette can be employed to deliver drugs to the neuron interior ( Evans & Marty, 1986). Recordings were made with allopregnanolone, or ganaxolone present in the recording pipette to explore whether the steroid could modulate synaptic GABA _A_Rs when delivered to the intracellular compartment. These recordings were compared to separate control recordings ( *i.e.* they were not paired). The presence of allopregnanolone in the recording pipette significantly increased the WT mIPSC τ _W_ in a concentration-dependent manner ( Table S14, Figure S3 A,C; *P* < 0.05). Here, the higher concentration of allopregnanolone (10 µM) had a comparatively large concentration-dependent effect on GABA _A_R mIPSCs when presented acutely within the recording electrode. This finding indicates that neurosteroids are able to exert their effect *via* the intracellular compartment and is in agreement with the literature ( Akk *et al.*, 2005).

The concentrations of pipette-applied allopregnanolone are relatively high, but the time scale from application to recording is short (< 10 minutes) and dialysis rate of the intracellular contents may be an influential limiting factor. The presence of ganaxolone in the recording pipette also significantly increased the WT mIPSC τ _W_ in a concentration-dependent manner ( Table S15, Figure S3 E,G, *P* < 0.05). The effect of pipette-applied ganaxolone is consistent with the recordings described above with allopregnanolone, although ganaxolone had a less pronounced effect on GABA _A_R mIPSCs. Collectively these experiments illustrate that steroid penetration of brain slice tissue is a significant limiting factor, the intracellular application is an effective method of presenting neuroactive steroids to GABA _A_Rs ( Table S14–Table S16, Figure 1, Figure S3 A–H) and that, in these neurons, allopregnanolone is a more effective modulator of the synaptic GABA _A_R than ganaxolone ( Figure S3 C,D,G,H).

Recordings were also made in *ob/ob* neurons with allopregnanolone, or ganaxolone, present in the recording pipette. The *ob/ob* mIPSC τ _W_ was increased by the presence of allopregnanolone in the recording pipette ( Table S14, Figure S3 B,C; *P* < 0.05). After normalisation, intracellular allopregnanolone 3 – 10 μM increased τ _W_ to similar percentages of the strain representative controls ( Table S14, Figure S3 D, *P* > 0.05). These findings are consistent with the hypothesis that there is no difference in the sensitivity of the cortical GABA _A_R to neurosteroids between the *ob/ob* and WT mice.

The *ob/ob* mIPSC τ _W_ was increased by the presence of ganaxolone in the recording pipette ( Table S15, Figure S3 F,G, *P* < 0.05). Ganaxolone did not have a concentration-dependent effect on *ob/ob* cortical GABA _A_R mIPSCs when acutely delivered intracellularly and the magnitude of the effect was less than that produced by equivalent pipette concentrations of allopregnanolone ( Table S14– Table S15, Figure S3 C,D,G,H,L, *P* > 0.05). There was no difference in the GABA _A_R mIPSC τ _W_ between the *ob/ob* and the WT when treated with intracellular ganaxolone. This finding is consistent with the recordings described above with allopregnanolone, although intracellular ganaxolone had a less pronounced effect.

In order to explore whether indometacin could be a silent competitive antagonist at the GABA _A_R (and therefore prevent modulation by ganaxolone) recordings were made with indometacin ± ganaxolone presented in the pipette. The WT mIPSC τ _W_ was unchanged by the presence of indometacin in the pipette and indometacin had no effect on the modulatory action of ganaxolone ( Table S16, Figure S3 I,J, *P* >0.05).

Recordings were also made in *ob/ob* neurons with indometacin ± ganaxolone presented in the pipette. The mIPSC τ _W_ in *ob/ob* mice was unaffected by the presence of indometacin in the pipette and indometacin had no effect on the modulatory action of ganaxolone ( Table S16, *P* >0.05). It is possible that indometacin may inhibit the action of ganaxolone by preventing its conversion to a more active compound (such as allopregnanolone).

The WT and *ob/ob* mIPSC τ _W_ was unaffected by the presence of DHP in the recording pipette ( Table S17, *P* > 0.05). These findings contrast with the significant effects of intracellular allopregnanolone and ganaxolone and are consistent with the idea that DHP is a precursor compound ( Figure 1, Figure S3 K,L). Considering these results it would be difficult to make the case for the alternative hypothesis that GABA _A_R sensitivity is increased in *ob/ob* and *db/db* mice.

### Behavioural work

The developmental age of P60–75 was chosen for both electrophysiological and behavioural experiments as it represents physiological maturity and facilitates a comparison with the *ob/ob* mouse model of T2DM, which develops hypersensitivity to pain by this age ( Drel *et al.*, 2006; Latham *et al.*, 2009).

### *ob/ob* mice have impaired sensorimotor coordination in comparison to WT mice

**Figure 6.**
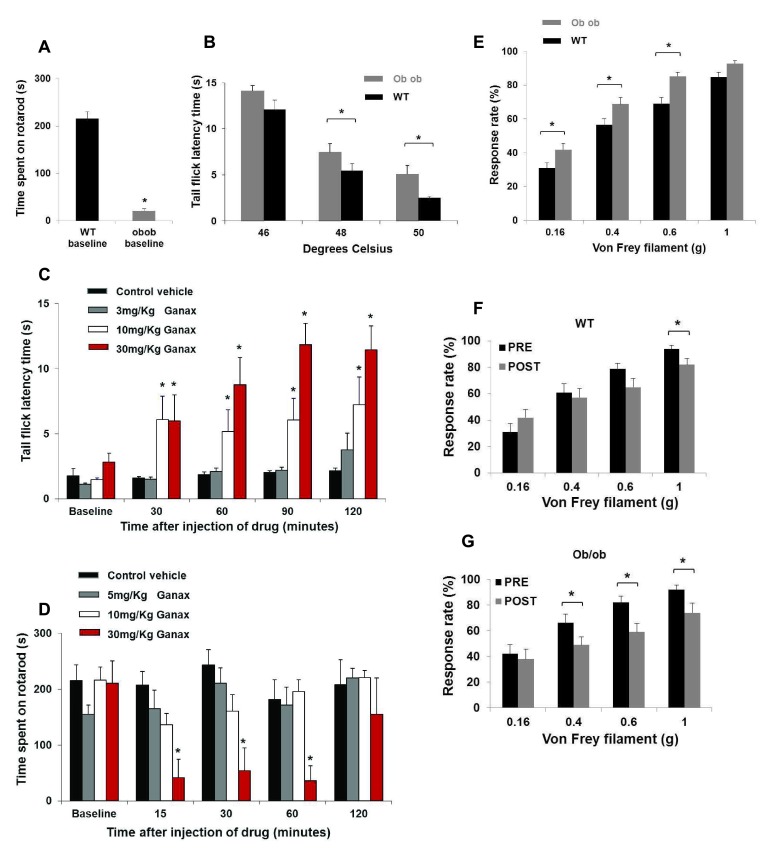
*ob/ob* mice have impaired sensorimotor coordination, exhibit hypoalgesia to noxious heat and mechanical hypersensitivity in comparison to WT mice. Ganaxolone impairs thermal nociception in WT mice at doses low enough to preserve rotarod performance. Ganaxolone reduces mechanical hypersensitivity in *ob/ob* mice and reduces mechanical nociceptive pain in both WT and *ob/ob* mice. ( **A**) Histogram illustrating the dramatic impairment of sensorimotor function exhibited by mature *ob/ob* mice. The *ob/ob* mice were able to remain on the accelerating rotarod for a significantly shorter time than were WT mice (n = 15; Mann-Whitney Rank Sum test *P* < 0.001). ( **B**) Histogram illustrating that mature *ob/ob* mice have a delayed response to thermal noxious stimuli in comparison to age-matched WT mice. ( *P* < 0.05). There was no significant difference at the less noxious temperature of 46°C (n = 20 per group, Mann-Whitney Rank Sum test *P* > 0.05). A maximum withdrawal latency of 15 seconds was enforced to minimise the possibility of tissue damage. ( **C**) Histogram illustrating that *ob/ob* mice exhibit mechanical hypersensitivity in comparison to WT mice. The *ob/ob* mice responded significantly more frequently than the WT mice to the 0.16 g, 0.4 g and 0.6 g filaments ( *P* < 0.05). By contrast, there was no significant difference in the frequency of responses to the 1 g vF filament (Mann-Whitney Rank Sum test *P* > 0.05). These results confirm the presence of mechanical hypersensitivity in the *ob/ob* mouse. ( **D**) Histogram illustrating that ganaxolone induced a dose-dependent prolongation of tail withdrawal latency in WT mice after 30 minutes ( *P* < 0.05). The solubilising vehicle β-CD had no effect (Kruskall Wallis One-Way ANOVA on ranks *P* > 0.05). ( **E**) Histogram showing that at 15, 30 and 60 minutes post-injection the highest dose of ganaxolone (30mg/kg) significantly impaired performance of WT mice on the rotarod ( *P* < 0.05). The effect of ganaxolone (30 mg/kg) was no longer apparent at 120 minutes post-injection ( *P* > 0.05). The solubilising vehicle β-CD had no effect on rotarod performance (Kruskall Wallis One-Way ANOVA on ranks *P* > 0.05). ( **F**) Histogram illustrating that ganaxolone had no impact on the response to the 0.16 g, 0.4 g or 0.6 g vF filaments in WT mice ( *P* > 0.05) but ganaxolone did reduce the response of WT mice to the 1.0 g vF filament (n = 10 per group, Wilcoxon signed rank test (before & after) *P* < 0.05). These data suggest that ganaxolone is analgesic for mechanical nociceptive pain in WT mice. WT mice do not exhibit hypersensitivity under normal conditions; therefore it is not unexpected that these drugs had no effect on the response to the smaller vF filaments. ( **G**) Histogram illustrating that ganaxolone had no impact on the response to the 0.16 g vF filament the *ob/ob* mouse ( *P* > 0.05). In contrast, ganaxolone reduced the response of the *ob/ob* mouse to the 0.4 g, 0.6 g and 1 g vF filaments (n = 10 per group, Wilcoxon signed rank test (before & after) *P* < 0.05). Ganaxolone reduced the response of the *ob/ob* mouse to the 0.4 g and the 0.6g vF filaments, which is consistent with the idea that this neuroactive steroid may reduce mechanical hypersensitivity. As described earlier, the 1.0 g vF filament induces a withdrawal response on ~85% of occasions in the WT and ~93% in the *ob/ob* mouse and may therefore be considered as an unambiguous test of mechanical nociceptive pain. Taken as a whole, these results show that ganaxolone was effective for mechanical nociceptive pain in both strains of mice.

The *ob/ob* mice were able to remain on the rotarod for a significantly shorter time than were WT mice (WT = 216 ± 14 s, n = 15; *ob/ob* = 21 ± 5 s, n = 15; Mann-Whitney Rank Sum test, *P* < 0.001; Figure 6A). The impaired rotarod performance of the *ob/ob* mouse is consistent with a previous report ( Mayers *et al.*, 2009). It is not possible to have obese WT mice as controls because those obese mice would be likely to have similar neurological consequences as the *ob/ob* mice.

### *ob/ob* mice exhibit prolonged tail withdrawal from noxious heat in comparison to WT mice

The tail flick test was used to characterise the response of the WT and the *ob/ob* mouse to three distinct temperatures. At 48°C and 50°C *ob/ob* mice exhibited significantly longer tail withdrawal latencies compared to WT mice ( Table S18, Figure 6B, *P* < 0.05). By contrast, there was no significant difference with a water temperature of 46°C ( Table S18, Figure 6B, *P* > 0.05). These findings are consistent with the phenomenon of thermal hypoalgesia in *ob/ob* mice reported by Drel *et al.* (2006), but conflict with the thermal hyperalgesia reported by Latham *et al.* (2009).

### Ganaxolone increases tail withdrawal latency in WT mice. Ganaxolone impairs thermal nociception in WT mice

The tail flick test is a useful tool for assessing thermal nociception. Ganaxolone was considered *a priori* considered to be a metabolically stable synthetic analogue of allopregnanolone, the molecular structure of which differs only by having an extra methyl group that prevents oxidation to an inactive form and has previously been used in clinical trials ( Carter *et al.*, 1997). Ganaxolone required to be solubilised with hydroxypropyl β-CD prior to intraperitoneal injection ( Besheer *et al.*, 2010; Carter *et al.*, 1997; Reddy & Rogawski, 2010). There were no significant differences in the baseline withdrawal latencies in response to a noxious thermal stimulus (50°C) between the four groups of mice used to examine the effects of ganaxolone ( Table S19, Figure 6C, *P* > 0.05). Ganaxolone induced a dose-dependent prolongation of tail withdrawal latency in WT mice after 30 minutes and this effect lasted more than 90 minutes ( Table S19, Figure 6C, *P* < 0.05). These data suggest that ganaxolone exhibits a dose-dependent analgesic effect in a test of thermal nociception in WT mice. This finding is consistent with the intrathecal administration of ganaxolone ( Asiedu *et al.*, 2012) and also other reports of the analgesic effects of similar neurosteroids in rats in the setting of post-chemotherapy neuropathy ( Meyer *et al.*, 2010; Meyer *et al.*, 2011).

### High doses of ganaxolone impair rotarod performance of WT mice

Only the highest dose of ganaxolone significantly impaired the performance of WT mice on the rotarod. There was no significant difference in the baseline rotarod performance of the four groups in the study ( Table S20, Figure 6D, *P* > 0.05). However, at 15, 30 and 60 minutes post-injection the highest dose of ganaxolone (30 mg/kg) significantly impaired performance of WT mice on the rotarod ( Table S20, Figure 6D, *P* < 0.05). The effect of ganaxolone (30 mg/kg) was no longer apparent at 120 minutes post-injection ( Table S20, Figure 6D, *P* > 0.05). Taken in conjunction with the tail flick data, these data suggest that ganaxolone exhibits an analgesic effect at a dose of 10 mg/kg but only impairs rotarod performance in WT mice at higher doses such as 30 mg/kg. In addition, the dose-dependent effect on rotarod performance is consistent with the literature ( Carter *et al.*, 1997).

### *ob/ob* mice exhibit mechanical hypersensitivity in comparison to WT mice

von Frey (vF) filaments have previously been employed *via* the up-down method to demonstrate that the *ob/ob* mechanical hypersensitivity by the age of P60 ( Drel *et al.*, 2006; Latham *et al.*, 2009). The 0.16 g, 0.4 g and 0.6 g vF filaments elicit withdrawal responses on 25%, 40% and 60% of occasions respectively in WT mice. If the *ob/ob* mouse has an exaggerated response to these filaments this would be consistent with mechanical hypersensitivity ( Merskey & Bogduk, 1994). The 1 g vF filament elicited a withdrawal response on approximately 90% of occasions, therefore it is considered to be a clear test of mechanical nociceptive pain in the WT mouse. This method was adapted from work published in rats with neuropathic sensitisation ( Meyer *et al.*, 2011). The *ob/ob* mice responded significantly more frequently than the WT mice to the 0.16 g, 0.4 g and 0.6 g filaments ( Table S21, Figure 6E, *P* < 0.05). By contrast, there was no significant difference in the frequency of responses to the 1 g vF filament ( Table S21, Figure 6E, *P* > 0.05). These results confirm the presence of mechanical hypersensitivity in the *ob/ob* mouse, which is consistent with reports in the literature ( Drel *et al.*, 2006; Latham *et al.*, 2009).

### Ganaxolone reduces mechanical hypersensitivity in *ob/ob* mice and reduces mechanical nociceptive pain in both WT and *ob/ob* mice

vF filaments were used for the comparison of mechanical sensitivity of WT and *ob/ob* mice before and after intraperitoneal drug administration. The β-CD vehicle had no impact on the response to any of the vF filaments in WT or *ob/ob* mice ( Table S22, *P* > 0.05). Ganaxolone (10 mg/kg) had no impact on the response to the 0.16 g, 0.4 g or 0.6 g vF filaments in the WT mouse ( Table S23, Figure 6F, *P* > 0.05) but ganaxolone did reduce the response of WT mice to the 1.0 g vF filament ( Table S23, Figure 6F, *P* < 0.05). These data suggest that ganaxolone is analgesic for mechanical nociceptive pain in WT mice. By definition, WT mice do not exhibit hypersensitivity or allodynia under normal conditions; therefore it is perhaps unsurprising that these drugs did not impact on the response to the smaller vF filaments.

Ganaxolone had no impact on the response rate to the 0.16 g vF filament in *ob/ob* mice ( Table S24, Figure 6G, *P* > 0.05), but in contrast, ganaxolone reduced the response rate of *ob/ob* mice to the 0.4 g, 0.6 g and 1 g vF filaments ( Table S24, Figure 6G, *P* < 0.05). *ob/ob* mice have exaggerated baseline response rates to the 0.16 g, 0.4 g and 0.6 g vF filaments in comparison to WT mice ( Figure 6E). Ganaxolone reduced the response rates of *ob/ob* mice to the 0.4 g and 0.6 g vF filaments, which is consistent with the idea that these neurosteroids may reduce mechanical hypersensitivity. As described earlier, the 1.0 g vF filament induces a withdrawal response on ~85% of occasions in WT mice and ~93% in *ob/ob* mice ( Figure 6E) and may therefore be considered as an unambiguous test of mechanical nociceptive pain. Taken as a whole, these results show that ganaxolone was effective for mechanical nociceptive pain in both strains of mice.

## Discussion

The decay time of GABA _A_R mIPSCs decreases with development at three levels of the pain pathway. GABA _A_Rs from pain pathway neurons are sensitive to modulation by neurosteroids, and this was explored using γ-CD applied within the recording pipette suggesting that neurosteroids may be synthesised within neurons themselves. The endogenous neurosteroid tone that previously appeared to be lost with maturation re-emerges in the adult mouse cortex, which is suggestive of a significant physiological role. Layer 2/3 pyramidal neurons of the somatosensory cortex are involved in the pain pathway, exhibit relatively homogenous GABA _A_R mIPSCs and are accessible for electrophysiological experimentation in mature animals. In contrast, there are inherent difficulties in studying neurons from other parts of the pain pathway in mature mice. Specifically, there is a relatively wide variation in decay time of GABA _A_R mIPSCs from Lamina II neurons of the spinal cord ( Mitchell *et al.*, 2007) making it very challenging to assess the effects of pharmacological agents on decay time. Separately, thalamic neurons of mice over the age of P25 have a high density of axonal projections that obscures visualisation of individual neurons ( Cox *et al.*, 1997; Pinault & Deschenes, 1998).

There are no published reports of the electrophysiological characterisation of synaptic GABA _A_R function for T2DM. It was unknown whether neurosteroid tone would be altered in mice with diabetic neuropathy. In inflammatory pain, neurosteroidogenesis is upregulated to mediate a form of endogenous analgesia by enhancing GABAergic neural inhibition ( Poisbeau *et al.*, 2005). The *ob/ob* model of T2DM is particularly useful for the study of neuropathic pain because it develops a more clinically relevant form of diabetes than other models ( Cefalu, 2006; Drel *et al.*, 2006; Latham *et al.*, 2009; Lindstrom, 2007; Sullivan *et al.*, 2007). However, it should be noted that no animal model of diabetes fully replicates the human phenotype. *ob/ob* mice are deficient of the hormone leptin and it was considered that leptin itself may modulate GABA _A_R function ( Solovyova *et al.*, 2009). Therefore, recordings were made in a second mouse model of T2DM, the *db/db* mouse, which is able to synthesise leptin but lacks the leptin receptor ( Bates *et al.*, 2005; Cefalu, 2006).

GABA _A_R mIPSCs from both *ob/ob* and *db/db* mice had significantly shorter decay times than the three WT strains, making it unlikely that leptin is involved. A decrease in the sensitivity of the GABA _A_R to allosteric modulators such as neurosteroids would be a possible explanation for the shorter GABA _A_R mIPSCs. However, reduced GABA _A_R sensitivity is inconsistent with the exaggerated effect of ganaxolone and DHP in *ob/ob* and the similar effect of allopregnanolone in both WT and diabetic cortical neurons. Pipette-applied allopregnanolone or ganaxolone induced the same concentration-dependent increase in mIPSC decay time, in *ob/ob* and WT, in keeping with Akk *et al.* (2005). A possible explanation may be that in T2DM mice there is a reduction in the endogenous neurosteroid tone, which is caused by a common pathological insult. Reduced neurosteroid tone could result in diminished GABAergic inhibition and subsequently a hypersensitive phenotype. Theoretically, other endogenous compounds that modulate the GABA _A_R such as endocannabinoids may be affected by a common mechanism in T2DM; however the selective effects of the neurosteroid scavenger γ-CD (in comparison to the lack of effect with α-CD and β-CD) make this less likely. γ-CD reduced the GABA _A_R mIPSC decay time to similar baseline values for all five lines of mice. These findings are consistent with the hypothesis that mature WT mice possess an endogenous autocrine neurosteroid tone that may fine-tune GABA _A_R function under physiological conditions but is reduced in *ob/ob* and *db/db* mice (in which underlying GABA _A_R function and sensitivity are otherwise unchanged). Thus, in neuropathic pain associated with diabetes, neurosteroid levels may be reduced, which contrasts to inflammatory pain, where neurosteroid levels may be upregulated ( Poisbeau *et al.*, 2005).

With reference to neurosteroidogenesis in the diabetic mice, incubation treatment with progesterone produced a modest prolongation of GABA _A_R mIPSC decay time in *ob/ob* mice that was similar to WT controls and was not concentration-dependent from 1–50 μM. Additional recordings made at the highest progesterone concentration in *db/db* mice were no different to those of the WT or *ob/ob.* This suggests that enzymatic function responsible for the endogenous synthesis of allopregnanolone is intact in *ob/ob* and *db/db*. Indeed, due to the shorter baseline decay time of mIPSCs of *ob/ob* and *db/db* mice, progesterone actually had a proportionately greater effect in the diabetic mice than in WT controls. In keeping with what had been observed in the WT, incubation treatment with finasteride (5α-R inhibitor) also had no effect on *ob/ob* mIPSCs directly, but was able to block the effect of progesterone in *ob/ob* mice.

Incubation treatment with DHP (the progesterone metabolite) produced a significant, concentration-dependent prolongation of GABA _A_R mIPSC decay time that was exaggerated in diabetic mice compared to WT. This finding suggests that not only is 3α-HSD enzymatic function preserved in mature T2DM mice, but it may actually be upregulated. A potential increase in 3α-HSD activity could be a partial compensation for a reduced baseline endogenous neurosteroid tone in diabetic mice. In keeping with what had been observed in WT mice, pipette-applied DHP had no effect on the *ob/ob* mIPSCs. The lack of effect of DHP is consistent with DHP’s role as a metabolic precursor to the active compound allopregnanolone.

Ganaxolone incubation treatment also had a greater impact in *ob/ob* mice compared with WT, despite the compound being considered previously to be more metabolically stable ( Carter *et al.*, 1997). This finding permits the possibility that ganaxolone may potentially be demethylated to allopregnanolone in order to exert maximal effect on cortical GABA _A_Rs. The idea of ganaxolone as both an agonist and precursor to allopregnanolone would also be consistent with the relatively larger effect of ganaxolone incubation in *ob/ob* mice, which may exhibit an upregulation of key enzymes such as CYP2C and 3α-HSD. The inhibitory effect of indometacin on ganaxolone incubation treatment is also consistent with the notion of ganaxolone as an active precursor of allopregnanolone. Indeed, indometacin had no impact on *ob/ob* cortical neurons when present in the pipette or after 2 hours of incubation treatment. When indometacin and ganaxolone were co-applied within the pipette, indometacin had no impact on the ability of ganaxolone to prolong mIPSCs. The CYP2C subfamily of enzymes is known to metabolise neurosteroids ( McFadyen *et al.*, 1998; Miksys & Tyndale, 2002) and cholesterol is metabolised to pregnenolone by CYP450scc ( Miksys & Tyndale, 2002; Schumacher *et al.*, 2012). Therefore, one possible explanation for this is that indometacin could compete with ganaxolone for the demethylating effects of CYP2C9, however, it must be noted that indometacin may have other non-specific effects.

The underlying mechanism responsible for the reduced neurosteroid tone in the diabetic mice is uncertain, but may be due to mitochondrial dysfunction ( Chowdhury *et al.*, 2013; Edwards *et al.*, 2008; Fernyhough *et al.*, 2010; Vincent *et al.*, 2010). It has been postulated that hyperglycaemia has several detrimental effects including the excessive donation of electrons to the mitochondrial electron transport chain, which induces an increased production of reactive oxygen species. The increased availability of electrons may lead to the partial reduction of oxygen to neurotoxic superoxide radicals ( Chowdhury *et al.*, 2013; Fernyhough *et al.*, 2010; Vincent *et al.*, 2010). Mitochondrial dysfunction could also account for both mechanical hypersensitivity associated with a reduction in mitochondrial-derived neurosteroids and also for thermal hyposensitivity associated with axonal degeneration ( Chowdhury *et al.*, 2013; Drel *et al.*, 2006; Latham *et al.*, 2009; Vincent *et al.*, 2010). The mechanisms of mitochondrial dysfunction in diabetic neuropathy has been covered by other authors ( Chowdhury *et al.*, 2013; Fernyhough *et al.*, 2010; Vincent *et al.*, 2010). These data are consistent with the possibility that neurosteroidogenic enzyme function may be upregulated in the *ob/ob* mice.

The behavioural work was consistent with the known phenotype of the *ob/ob* ( Drel *et al.*, 2006; Mayers *et al.*, 2009) and translated the electrophysiological findings into measurable anti-nociceptive effects. The polyneuropathy phenotype with seemingly paradoxical mechanical hypersensitivity and thermal hyposensitivity is in fact consistent with the progressive diabetic neuropathy observed in the literature ( Cefalu, 2006; Drel *et al.*, 2006; Kaplan & Wagner, 2006; Latham *et al.*, 2009). The mechanical hypersensitivity of *ob/ob* mice and the increased response to ganaxolone reflected the observed electrophysiological findings. *Ob/ob* mice developed obesity and T2DM on a normal diet, which led to painful neuropathy by the age of P60-75. At this age they exhibited sensorimotor impairment, thermal hypoalgesia, cold allodynia and mechanical allodynia. In WT mice, the neuroactive steroid ganaxolone impaired sensorimotor function at 30 mg/kg, but the lower dose of 10 mg/kg did not, and was analgesic for thermal and mechanical nociceptive pain. The effect of ganaxolone on thermal nociception and sensorimotor impairment could not be tested on *ob/ob* mice due to their pre-existing deficits. However, ganaxolone significantly reduced mechanical allodynia in *ob/ob* mice. These results suggest that GABA _A_R-modulatory neurosteroidal drugs such as ganaxolone may have analgesic properties for nociceptive pain and also neuropathic pain by restoration of the depressed endogenous neurosteroid tone.

## Data and software availability

Open Science Framework: Dataset of ‘Neurosteroids are reduced in diabetic neuropathy and may be associated with the development of neuropathic pain’, doi: 10.17605/osf.io/bk3tw ( Humble, 2016).

Analysed raw data can be found in the supplementary tables (See Supplementary material).

Please refer to Methods section for details of standard software used for data analysis.
