# Supplementary material for: Neurosteroids are reduced in diabetic neuropathy and may be associated with the development of neuropathic pain
Source: F1000Res. 2016 Aug 5;5:1923. [Version 1] doi: 10.12688/f1000research.9034.1 (PMC5345788; doi:10.12688/f1000research.9034.1)
Supplement: Supplementary file 1 [file f1000research-5-9720-s0000.tgz › 4d35a57f-65e6-4c88-939f-67e57d9a52a4.docx]

**Additional material for: Neurosteroids are reduced in diabetic neuropathy and may be associated with the development of neuropathic pain**

**GABA_A_Rs from pain pathway neurons are sensitive to modulation by neurosteroids**

The neurosteroid allopregnanolone (3α-hydroxy-5α-pregnan-20-one) and a closely related synthetic structural analogue ganaxolone (3β-methyl-3α-ol-5α-pregnan-20-one) have proven efficacy in enhancing the function of synaptic GABA_A_Rs in other regions of the central nervous system such as the dentate gyrus and the rat spinal cord (Belelli and Herd 2003; Hosie *et al.,* 2006; Mitchell *et al.,* 2007). The influence of the neurosteroid on the mIPSCs of LII neurons was investigated at two developmental stages: P8-11 and P17-25. Previous studies have revealed this to be a sensitive parameter (Mitchell *et al.,* 2007). Recordings were made before and after the bath application of neurosteroids. The compounds were bath-applied after at least four minutes of stable recording enabling the comparison of paired recordings. For P8-11 neurons the mean τ_W_ was increased by 22 ± 6 % (n = 7) by 1μM allopregnanolone after ten minutes. By contrast, for P17-25 neurons the steroid appeared more effective, increasing τ_W_ by 97 ± 47% (P8-11, control = 20 ± 1.7ms, 1μM allopregnanolone = 24.5 ± 2.4ms, n = 7, Student’s paired t test, *P* <0.05; P17-25, control = 21.5 ± 5ms, allopregnanolone = 39.8 ± 9ms, n = 6, Student’s paired t test, *P* <0.05). The acute application of ganaxolone 1μM also induced a prolongation of the exponential decay time of mIPSCs in nRT neurons (τ_W_: P17-25, control = 15.2 ± 1.2 ms, n = 6; ganaxolone 1μM = 18.2 ± 1.8 ms, n = 6; Student’s paired t test, *P* <0.05). The acute application of allopregnanolone 1μM induced a modest prolongation of exponential decay time of GABA_A_R mIPSCs of mature cortical neurons (τ_W_: P60-75, control = 3.9 ± 0.3 ms, n = 6; allopregnanolone 1μM = 4.4 ± 0.4 ms, n = 6; Paired Student’s t test, *P* <0.05). The acute application of ganaxolone 1μM also induced a modest prolongation of the mIPSC exponential decay time of mature cortical neurons (τ_W_: P60-75, control = 4 ± 0.2 ms, n = 6; ganaxolone 1μM = 4.7 ± 0.2 ms, n = 6; Student’s paired t test, *P* <0.05). When the data for τ_W_ was normalised for comparison, there was no significant difference between allopregnanolone and ganaxolone at P60-75 (Two-way RM ANOVA, *P* >0.05). The acute application of a 3-fold greater concentration of ganaxolone (3μM) produced a similar prolongation of the mIPSC exponential decay time to that caused by 1µM of this steroid (τ_W_: control = 3.5 ± 0.2ms, n = 4; ganaxolone 3μM = 4.2 ± 0.2ms, n = 4; Student’s paired t test, *P* <0.05). When the data for τ_W_ was normalised for comparison, there was no significant difference between ganaxolone 1μM and 3μM at P60-75 (Two-way RM ANOVA, *P* >0.05).

**The effect of γ-cyclodextrin (γ-CD) applied by three different methods on nRT GABA_A_R mIPSCs (P6-7)**

γ-CD is a barrel-shaped molecule known to sequester neurosteroids (Shu *et al.,* 2004;2007) therefore this molecule may be used as an experimental tool. Recordings were made from P6-7 nRT neurons using three methods of application of γ-CD to confirm which method was optimal. The γ-CD was presented within the recording electrode (intracellularly), applied *via* the extracellular solution, or by inclusion in the pipette, in the extracellular recording solution and in the incubation chamber containing the brain slice preparation prior to recording (*i.e.* 2 hours of preincubation). When γ-CD (1mM) was present in the extracellular solution (ECS) for at least 5 minutes, the mIPSC decay (τ_W_) was not decreased significantly (P6-7 control = 31.1 ± 2 ms, n = 6, P6-7 γ -CD (ECS) = 28.7 ± 2.8 ms, n = 6; Student’s paired t test, *P* <0.05). When the γ-CD was present both intracellularly (0.5mM), extracellularly (1mM) and had previously been incubated with the brain slice preparation in the holding chamber (1mM) *i.e.* the “triple treatment”, the mIPSC decay (τ_W_) was significantly decreased. However, this protocol was no more effective than when the γ-CD was present only intracellularly (P6-7 control = 33.2 ± 1 ms, n = 24, P6-7 γ-CD [“triple treatment”] = 23.7 ± 1 ms, n = 13; Student’s unpaired t test, *P* < 0.05). These results indicate that intracellular application of γ-CD *via* the recording pipette is the optimal method of application and is consistent with the hypothesis that the GABA_A_R-active neurosteroids are synthesised within the pain pathway neurons themselves (Akk *et al.,* 2005; Chisari *et al.,* 2009; Tsutsui *et al.,* 2008).

**The effect of γ-CD 0.5mM in the recording pipette on nRT GABA_A_R-mediated mIPSCs at three stages of development**

Recordings were made at three stages of development in order to explore fluctuations in the endogenous neurosteroid tone of nRT neurons from P6–P25. γ-CD 0.5mM was present only within the recording electrode. Note in this study recordings were made and compared from a population of control and γ-CD treated (in the recording pipette) nRT neurons *i.e.* the recordings were not paired. The mIPSC decay (τ_W_) of P6-7 nRT neurons was decreased by the presence of γ-CD (P6-7 control = 33.2 ± 1 ms, n = 24, P6-7 γ-CD = 21.6 ± 1.4 ms, n = 8; Student’s unpaired t test, *P* <0.01). The mIPSC decay (τ_W_) of P10 nRT neurons was not decreased significantly by the presence of γ-CD (P10 control = 22.5 ± 0.7 ms, n = 14, P10 γ-CD = 22.1 ± 0.6 ms, n = 13; Student’s unpaired t test, < 0.05). The mIPSC decay (τ_W_) of P17-25 nRT neurons was not decreased by the presence of γ-CD (P17-25 control = 18.2 ± 0.6 ms, n = 21, P17-25 γ-CD = 19 ± 1 ms, n = 15; Student’s unpaired t test, >0.05).
