# Supplementary material for: Neurosteroids are reduced in diabetic neuropathy and may be associated with the development of neuropathic pain
Source: F1000Res. 2016 Aug 5;5:1923. [Version 1] doi: 10.12688/f1000research.9034.1 (PMC5345788; doi:10.12688/f1000research.9034.1)
Supplement: Supplementary file 2 [file f1000research-5-9720-s0001.tgz › f446b999-579c-472d-a614-fc04bdcf0009.doc]

Table S1. Pipette applied γ-cyclodextrin (CD) reduces decay time of GABA_A_ mIPSCs of layer 2/3 cortical neurones at two stages of development (P9-10 and P60-75)

|  | **P9-10**  **Control**  **(n=8)** | **P9-10**  **γ-CD**  **(n=6)** | **P60-75**  **Control**  **(n=35)** | **P60-75**  **γ-CD**  **(n=15)** |
| --- | --- | --- | --- | --- |
| **Peak amplitude (pA)** | -36 ± 2 | -35 ± 3 | -59 ± 2 | -58 ± 2 |
| **Rise Time (ms)** | 0.7 ± 0.1 | 0.7 ± 0.1 | 0.4 ± 0.1 | 0.4 ± 0.1 |
| **Charge transfer (fC)** | -333± 20 | *-266 ± 22 | -242 ± 9 | *-185 ± 8 |
| **T70 (ms)** | **10.3 ± 0.3** | ***8.7 ± 0.6** | **5.2 ± 0.2** | ***3.7 ± 0.2** |
| **Tau w (ms)** | **10.0 ± 0.6** | ***7.4 ± 0.4** | **4.0 ± 0.1** | ***2.9 ±0.1** |
| **Frequency (Hz)** | 3 ± 0.5 | *1.6 ± 0.5 | 17 ± 2 | 18 ± 2 |

**P* < 0.05; unpaired Student’s t test, in comparison to age-matched control

Table S2. The effect of intracellular α/β/γ – cyclodextrin (CD) 0.5mM on GABA_A_R mIPSCs of layer 2/3 cortical neurones (P60-75) of WT and *ob/ob* mice

|  | **WT**  **Control**  **( n=35)** | **WT**  **α-CD**  **( n=7)** | **WT**  **β-CD**  **( n=6)** | **WT**  **γ-CD**  **( n=15)** | ***Ob/ob***  **(n=25)** | ***Ob/ob***  **α-CD**  **( n=5)** | ***Ob/ob***  **β-CD**  **( n=6)** | ***Ob/ob***  **γ-CD**  **( n=6)** |
| --- | --- | --- | --- | --- | --- | --- | --- | --- |
| **Peak amp (pA)** | -59 ± 2 | -55 ± 3 | -56 ± 3 | -58 ± 2 | -66 ± 2 | -65 ± 3 | -57 ± 3 | -71 ± 5 |
| **Rise Time (ms)** | 0.4 ± 0.1 | 0.5 ± 0.1 | 0.4 ± 0.1 | 0.4 ± 0.1 | 0.4 ± 0.1 | 0.4 ± 0.1 | 0.4 ± 0.1 | 0.4 ± 0.1 |
| **Charge transfer (fC)** | -242 ± 9 | -243 ± 15 | -261 ± 16 | **-185 ± 8 | -255 ± 12 | -260 ± 16 | -222 ± 14 | -225 ± 18 |
| **T70 (ms)** | **5.2 ± 0.2** | **5.7 ± 0.6** | **5.9 ± 0.5** | ****3.7 ± 0.2** | **4.7 ± 0.1** | **4.8 ± 0.2** | **4.6 ± 0.2** | ****3.8 ± 0.2** |
| **Tau w (ms)** | **4.0 ± 0.1** | **4.1 ± 0.5** | **4.5 ± 0.4** | ****2.9 ±0.1** | **3.5 ± 0.1** | **3.7 ± 0.2** | **3.6 ± 0.2** | ****3.0 ± 0.2** |
| **Frequency (Hz)** | 17 ± 2 | 18 ± 4 | 18 ± 5 | 18 ± 2 | 21 ± 3 | 17 ± 3 | 20 ± 4 | 19 ± 3 |

***P* < 0.05; one-way ANOVA (with *Post hoc* Newman Keul’s test), in comparison to strain-matched control (P60-75)

Table S3. *ob/ob* and *db/db* mice (P60-75) both had significantly greater body weights than the respective WT animals of the same age

|  | **C57/BL6**  **WT**  **(n=32)** | **B6.V-*Lep^+^***  **WT**  **(n=49)** | **BKS.Cg-*Dock7^m^+* WT**  **(n=5)** | ***ob/ob***  **(n=50)** | ***db/db***  **(n=50)** |
| --- | --- | --- | --- | --- | --- |
| **Body weight (g)** | 26.8 ± 0.3 | 24.7 ± 0.3 | 22.8 ± 1.0 | **35.6 ± 0.4 | **42.2 ± 0.8 |

***P* < 0.05, One-way ANOVA; *post hoc* Newman Keul’s test revealed significant differences between both diabetic mice and all three strains of WT mice, *P* < 0.05

Table S4. A comparison of synaptic GABA_A_R mIPSCs of cortical layer 2/3 neurones in adult WT, *ob/ob* and *db/db* mice.

|  | **WT**  **C57 BL6**  **Control**  **(n=35)** | **WT**  **B6.V-*Lep^+^/* OlaHsd**  **(n=18)** | **WT**  **BKS.Cg- *Dock7^m^+***  **(n=8)** | ***Ob/ob***  **(n=25)** | ***Db/db***  **(n=18)** |
| --- | --- | --- | --- | --- | --- |
| **Peak amp (pA)** | -59 ± 2 | -64 ± 2 | -74 ± 5 | -66 ± 2 | -60 ± 2 |
| **Rise Time (ms)** | 0.4 ± 0.1 | 0.5 ±0.1 | 0.4 ± 0.1 | 0.4 ± 0.1 | 0.4 ± 0.1 |
| **Charge transfer (fC)** | -242 ± 9 | -265 ±11 | -333 ± 26 | -255 ± 12 | -223 ± 8 |
| **T70 (ms)** | **5.2 ± 0.2** | **5.3 ± 0.2** | **5.6 ± 0.4** | ***4.7 ± 0.1** | ***4.6 ± 0.1** |
| **Tau w (ms)** | **4.0 ± 0.1** | **3.9 ± 0.2** | **4.3 ± 0.3** | ***3.5 ± 0.1** | ***3.5 ± 0.1** |
| **Frequency (Hz)** | 17 ± 2 | 24 ± 2 | 14.8 ± 2 | 21 ± 3 | 19 ± 3 |

**P* < 0.05; unpaired Student’s t test, in comparison to appropriate strain-matched control (P60-75)

Table S5. A comparison of the effects of 0.5mM γ-cyclodextrin (CD) on cortical GABA_A_R mIPSCs of adult WT, *ob/ob* and *db/db* mice (P60-75)

|  | **WT**  **C57 BL6**  **Control**  **γ-CD**  **(n=15)** | **WT**  **B6.V-*Lep^+^/* OlaHsd**  **γ-CD**  **(n=11)** | **WT**  **BKS.Cg- *Dock7^m^+***  **γ-CD**  **(n=5)** | ***Ob/ob***  **γ-CD**  **(n=6)** | ***Db/db***  **γ-CD**  **(n=9)** |
| --- | --- | --- | --- | --- | --- |
| **Peak amp (pA)** | -58 ± 2 | -55 ± 1 | -58 ± 3 | **-71 ± 5 | -54 ± 2 |
| **Rise Time (ms)** | 0.4 ± 0.1 | 0.4 ± 0.1 | 0.4 ± 0.1 | 0.4 ± 0.1 | 0.4 ± 0.1 |
| **Charge transfer (fC)** | -185 ± 8 | -175 ± 7 | -214 ± 19 | -225 ± 18 | -170 ± 11 |
| **T70 (ms)** | **3.7 ± 0.2** | **3.8 ± 0.2** | **4.4 ± 0.2** | **3.8 ± 0.2** | **3.9 ± 0.1** |
| **Tau w (ms)** | **2.9 ± 0.1** | **3.0 ± 0.1** | **3.3 ± 0.2** | **3.0 ± 0.2** | **2.9 ± 0.1** |
| **Frequency (Hz)** | 18 ± 2 | *25 ± 3 | 12 ± 1 | 19 ± 3 | 13 ± 3 |

Table S6. The effect of allopregnanolone (Allo) incubation treatment on GABA_A_R mIPSCs of L2/3 cortical neurones (P60-75) of WT, *ob/ob* and *db/db* mice

|  | **WT**  **Control**  **( n=35)** | ***Ob/ob***  **(n=25)** | ***Db/db***  **(n=18)** | **WT**  **100nM Allo**  **( n=9)** | ***Ob/ob***  **100nM Allo**  **( n=6)** | **WT**  **300nM Allo**  **( n=9)** | ***Ob/ob***  **300nM Allo**  **( n=8)** | ***Db/db***  **300nM Allo**  **( n=6)** |
| --- | --- | --- | --- | --- | --- | --- | --- | --- |
| **Peak amp (pA)** | -59 ± 2 | -66 ± 2 | -60 ± 2 | **-78 ± 2 | **-81 ± 2 | **-87 ± 5 | **-94 ± 4 | *-99 ± 3 |
| **Rise Time (ms)** | 0.4 ± 0.1 | 0.4 ± 0.1 | 0.4 ± 0.1 | 0.5 ± 0.1 | 0.5 ± 0.1 | 0.5 ± 0.1 | 0.5 ± 0.1 | 0.5 ± 0.1 |
| **Charge transfer (fC)** | -242 ± 9 | -255 ± 12 | -223 ± 8 | **-932 ± 84 | **-987 ± 82 | **-1489 ± 136 | **-1540 ± 100 | *-1604 ± 81 |
| **T70 (ms)** | **5.2 ± 0.2** | **4.7 ± 0.1** | **4.6 ± 0.1** | ****18.6 ± 1** | ****18.7 ± 1** | ****27 ± 1** | ****25.8 ± 0.9** | ***25.4 ± 0.6** |
| **Tau w (ms)** | **4.0 ± 0.1** | **3.5 ± 0.1** | **3.5 ± 0.1** | ****12.5 ± 1** | ****12.8 ± 1** | ****19 ± 1** | ****18.8 ± 1** | ***18.6 ± 0.6** |
| **Frequency (Hz)** | 17 ± 2 | 21 ± 3 | 19 ± 3 | 15 ± 3 | **10 ± 1 | **11 ± 1 | **11 ± 2 | 13 ± 2 |

***P* < 0.05; One-way ANOVA (with *Post hoc* Newman Keul’s test) in comparison to strain-matched control (P60-75)

Table S7. The effect of ganaxolone (Ganax) incubation treatment (300nM, 1μM or 3μM) on GABA_A_R mIPSCs of L2/3 cortical neurones (P60-75) of WT, *ob/ob* and *db/db* mice

|  | **WT**  **Control**  **( n=35)** | ***Ob/ob***  **(n=25)** | ***Db/db***  **(n=18)** | **WT**  **300nM**  **Ganax**  **( n=10)** | ***Ob/ob***  **300nM Ganax**  **( n=9)** | ***Db/db***  **300nM Ganax**  **( n=4)** | **WT**  **1μM Ganax**  **( n=10)** | ***Ob/ob***  **1μM Ganax**  **( n=10)** | ***Db/db***  **1μM Ganax**  **( n=8)** | **WT**  **3μM Ganax**  **( n=8)** |
| --- | --- | --- | --- | --- | --- | --- | --- | --- | --- | --- |
| **Peak amp (pA)** | -59 ± 2 | -66 ± 2 | -60 ± 2 | **-82 ± 6 | **-80 ± 7 | **-79 ± 8 | **-97 ± 9 | **-126 ± 5 | **-117 ± 3 | **-153 ± 6 |
| **Rise Time (ms)** | 0.4 ± 0.1 | 0.4 ± 0.1 | 0.4 ± 0.1 | 0.5 ± 0.1 | 0.5 ± 0.1 | 0.5 ± 0.1 | 0.6 ± 0.1 | 0.5 ± 0.1 | 0.6 ± 0.1 | 0.6 ± 0.1 |
| **Charge transfer (fC)** | -242 ± 9 | -255 ± 12 | -223 ± 8 | **-749  ± 88 | **-1104  ± 178 | **-1004  ± 81 | **-1575  ± 195 | **-2267  ± 162 | **-2193  ± 83 | **-4396  ± 268 |
| **T70 (ms)** | **5.2 ± 0.2** | **4.7 ± 0.1** | **4.6 ± 0.1** | ****13.4 ± 1** | ****20.7 ± 2** | ****19.3**  **± 0.7** | ****24.2 ± 1** | ****27.5 ± 1** | ****28.5**  **± 1.4** | ****44.2 ± 2** |
| **Tau w (ms)** | **4.0 ± 0.1** | **3.5 ± 0.1** | **3.5 ± 0.1** | ****10.9 ± 1** | ****15.4 ± 1** | ****14.8**  **± 0.4** | ****18.8 ± 1** | ****20.6 ± 1** | ****22 ± 1.6** | ****32.5 ± 1** |
| **Frequency (Hz)** | 17 ± 2 | 21 ± 3 | 19 ± 3 | 20 ± 3 | 13 ± 2 | 10 ± 2 | 13.5 ± 2 | 12 ± 1 | 10 ± 2 | **8 ± 1 |

***P* < 0.05; One-way ANOVA (with *Post hoc* Newman Keul’s test) in comparison to strain-matched control (P60-75)

Table S8. The effect of progesterone (Prog) incubation treatment on GABA_A_R mIPSCs of layer 2/3 cortical neurones (P60-75) of WT, *ob/ob* or *db/db* mice

|  | **WT**  **Control**  **( n=35)** | **WT**  **1μM**  **Prog**  **( n=6)** | **WT**  **3μM**  **Prog**  **( n=8)** | **WT**  **10μM Prog**  **( n=9)** | **WT**  **50μM**  **Prog**    **(n=9)** | ***Ob/ob***  **(n=25)** | ***Ob/ob***  **1μM**  **Prog**  **(n=5)** | ***Ob/ob***  **3μM**  **Prog**  **(n=10)** | ***Ob/ob***  **10μM**  **Prog**    **( n=7)** | ***Ob/ob***  **50μM**  **Prog**    **(n=12)** | ***db/db***  **50μM**  **Prog**    **(n = 6)** |
| --- | --- | --- | --- | --- | --- | --- | --- | --- | --- | --- | --- |
| **Peak amp (pA)** | -59 ± 2 | -65 ± 2 | -64 ± 3 | **-70 ± 2 | **-91 ± 5 | -66 ± 2 | -68 ± 5 | -78 ± 3 | -79 ± 3 | **-84 ± 4 | -79 ± 4 |
| **Rise Time (ms)** | 0.4 ± 0.1 | 0.5 ± 0.1 | 0.5 ± 0.1 | 0.5 ± 0.1 | 0.5 ± 0.1 | 0.4 ± 0.1 | 0.4 ± 0.1 | 0.5 ± 0.1 | 0.4 ± 0.1 | 0.5 ± 0.1 | 0.5 ± 0.1 |
| **Charge transfer** | -242 ± 9 | **-319 ± 19 | **-339 ± 26 | **-373 ± 20 | **-515 ± 34 | -255 ± 12 | **-352 ± 34 | **-409 ± 24 | **-474 ± 31 | **-499 ± 24 | -446 ± 24 |
| **T70 (ms)** | **5.2 ± 0.2** | ****6.5 ± 0.5** | ****6.8 ± 0.2** | ****7.3 ± 0.4** | ****7.6 ± 0.2** | **4.7 ± 0.1** | ****6.8 ± 0.7** | ****6.9 ± 0.2** | ****8.4 ± 0.6** | ****8.1 ± 0.4** | **7.8 ± 0.3** |
| **Tau w (ms)** | **4.0 ± 0.1** | ****4.7 ± 0.3** | ****5.2 ± 0.2** | ****5.1 ± 0.3** | ****5.3 ± 0.2** | **3.5 ± 0.1** | ****5.1 ± 0.3** | ****5.1 ± 0.1** | ****6.1 ± 0.4** | ****5.9 ± 0.3** | **5.5 ± 0.2** |
| **Frequency (Hz)** | 17 ± 2 | 16 ± 2 | 17 ± 2 | 19 ± 2 | **31 ± 3 | 21 ± 3 | 1 ± 2 | **27 ± 3 | 17 ± 4 | **29 ± 3 | 25 ± 4 |

***P* < 0.05; One-way ANOVA (with *Post hoc* Newman Keul’s test) in comparison to strain-matched control (P60-75)

Table S9. The effect of DHP incubation treatment on GABA_A_R mIPSCs of layer 2/3 cortical neurones (P60-75) of WT, *ob/ob* and *db/db* mice

|  | **WT**  **Control**  **( n=35)** | **WT**  **1μM DHP**  **( n=14)** | **WT**  **3μM DHP**  **(n=9)** | ***Ob/ob***  **(n=25)** | ***Ob/ob***  **1μM DHP**  **(n=18)** | ***Ob/ob***  **3μM DHP**  **(n=10)** | ***Db/db***  **(n=18)** | ***Db/db***  **3μM DHP**  **( n=8)** |
| --- | --- | --- | --- | --- | --- | --- | --- | --- |
| **Peak amp (pA)** | -59 ± 2 | **-73 ± 4 | **-69 ± 5 | -66 ± 2 | **-75 ± 3 | -70 ± 3 | -60 ± 2 | *-83 ± 3 |
| **Rise Time (ms)** | 0.4 ± 0.1 | 0.4 ± 0.1 | 0.5 ±0.1 | 0.4 ± 0.1 | 0.4 ± 0.1 | 0.5 ± 0.1 | 0.4 ± 0.1 | 0.5 ± 0.1 |
| **Charge transfer (fC)** | -242 ± 9 | **-433 ± 31 | **-646 ± 33 | -255 ± 12 | **-792 ± 63 | **-825 ± 62 | -223 ± 8 | *-1034 ± 57 |
| **T70 (ms)** | **5.2 ± 0.2** | ****8 ± 0.4** | ****13.9 ± 1** | **4.7 ± 0.1** | ****15.6 ± 1** | ****17.6 ± 0.8** | **4.6 ± 0.1** | ***19.1 ± 0.6** |
| **Tau w (ms)** | **4.0 ± 0.1** | ****5.9 ± 0.2** | ****10.2 ± 1** | **3.5 ± 0.1** | ****10.9 ± 0.6** | ****12.3 ± 0.7** | **3.5 ± 0.1** | ***13.4 ± 0.6** |
| **Frequency (Hz)** | 17 ± 2 | 16 ± 2 | 19 ± 6 | 21 ± 3 | 13 ± 1 | 14 ± 0.6 | 19 ± 3 | 16 ± 2 |

***P* < 0.05; One-way ANOVA (with *Post hoc* Newman Keul’s test) in comparison to strain-matched control (P60-75)

Table S10. Finasteride (Finast) prevents the effect of progesterone (Prog) incubation treatment on GABA_A_R mIPSCs of layer 2/3 cortical neurones (P60-75) of WT, *ob/ob* or *db/db* mice

|  | **WT**  **Control**  **(n=35)** | **WT**  **50μM Finast**    **(n=7)** | **WT**  **50μM Prog**  **(n=9)** | **WT**  **50μM Prog**  **& 50μM Finast**  **(n=7)** | ***Ob/ob***  **(n=25)** | ***Ob/ob***  **50μM Prog**    **(n=12)** | ***Ob/ob***  **50μM Finast**    **(n=6)** | ***Ob/ob***  **50μM Prog**  **& 50μM Finast**  **(n=6)** |
| --- | --- | --- | --- | --- | --- | --- | --- | --- |
| **Peak amp (pA)** | -59 ± 2 | **-67 ± 3 | **-91 ± 5 | -64 ± 3 | -66 ± 2 | **-84 ± 4 | -58 ± 3 | -65 ± 3 |
| **Rise Time (ms)** | 0.4 ± 0.1 | 0.4 ± 0.1 | 0.5 ± 0.1 | 0.4 ± 0.1 | 0.4 ± 0.1 | 0.5 ± 0.1 | 0.4 ± 0.1 | 0.4 ± 0.1 |
| **Charge transfer (fC)** | -242 ± 9 | **-299 ± 19 | **-515 ± 34 | -269 ± 10 | -255 ± 12 | **-499 ± 24 | -233 ± 18 | -232 ± 16 |
| **T70 (ms)** | **5.2 ± 0.2** | **5.7 ± 0.2** | ****7.6 ± 0.2** | **5.7 ± 0.1** | **4.7 ± 0.1** | ****8.1 ± 0.4** | **5.0 ± 0.2** | **4.9 ± 0.2** |
| **Tau w (ms)** | **4.0 ± 0.1** | **4.2 ± 0.1** | ****5.3 ± 0.2** | **4.1 ± 0.1** | **3.5 ± 0.1** | ****5.9 ± 0.3** | **3.7 ± 0.2** | **3.4 ± 0.2** |
| **Frequency (Hz)** | 17 ± 2 | **23 ± 2 | **31 ± 3 | 19 ± 1.5 | 21 ± 3 | *29 ± 3 | 13 ± 3 | 12 ± 2 |

***P* < 0.05; One-way ANOVA (with *Post hoc* Newman Keul’s test), in comparison to strain-matched control

Table S11. The 3α-HSD inhibitor provera reduces the effect of DHP but not ganaxolone (Ganax) incubation treatment on GABA_A_R mIPSCs of layer 2/3 cortical neurones (P60-75) of WT mice

|  | **WT**  **Control**  **(n=35)** | **WT**  **1uM**  **Provera**  **( n=6)** | **WT**  **3μM**  **DHP**  **(n=9)** | **WT**  **Provera & 3uM DHP**  **(n=7)** | **WT**  **300nM Ganax**  **(n=10)** | **WT**  **Provera & 300nM Ganax**  **(n=7)** |
| --- | --- | --- | --- | --- | --- | --- |
| **Peak amplitude (pA)** | -59 ± 2 | -66 ± 5 | -69 ± 5 | -61 ± 3 | -82 ± 6 | -75 ± 4 |
| **Rise Time (ms)** | 0.4 ± 0.1 | 0.5 ± 0.1 | 0.5 ±0.1 | 0.4 ± 0.1 | 0.5 ± 0.1 | 0.5 ± 0.1 |
| **Charge transfer (fC)** | -242 ± 9 | -310 ± 23 | -646 ± 33 | **-327 ± 19 | -749 ± 88 | -778 ± 46 |
| **T70 (ms)** | **5.2 ± 0.2** | **6.3 ± 0.3** | **13.9 ± 1** | **** 6.9 ± 0.1** | **13.4 ± 1** | **15.5 ± 0.7** |
| **Tau w (ms)** | **4.0 ± 0.1** | **4.6 ± 0.2** | **10.2 ± 1** | **** 5.2 ± 0.1** | **10.9 ± 1** | **10.9 ± 0.7** |
| **Frequency (Hz)** | 17 ± 2 | 23 ± 4 | 19 ± 6 | 16 ± 3 | 20 ± 3 | 17 ± 3 |

***P* < 0.05; One-way ANOVA (with *Post hoc* Newman Keul’s test) in comparison to 3μM DHP

Table S12. The 3α-HSD inhibitor indomethacin (indo) reduces the effect of DHP incubation treatment on GABA_A_R mIPSCs of layer 2/3 cortical neurones (P60-75) of WT mice

|  | **WT**  **Control**  **(n=35)** | **WT**  **30μM Indo**  **(n=6)** | **WT**  **3μM DHP**  **(n=9)** | **WT**  **3μM DHP & 30μM Indo**  **(n=6)** |
| --- | --- | --- | --- | --- |
| **Peak amplitude (pA)** | -59 ± 2 | -53 ± 2 | -69 ± 5 | **-56 ± 4 |
| **Rise Time (ms)** | 0.4 ± 0.1 | 0.4 ± 0.1 | 0.5 ±0.1 | 0.4 ± 0.1 |
| **Charge transfer (fC)** | -242 ± 9 | -222 ± 6 | -646 ± 33 | **-263 ± 23 |
| **T70 (ms)** | **5.2 ± 0.2** | **5.1 ± 0.2** | **13.9 ± 1** | ****5.8 ± 0.3** |
| **Tau w (ms)** | **4.0 ± 0.1** | **4.6 ± 0.2** | **10.2 ± 1** | **** 5.0 ± 0.3** |
| **Frequency (Hz)** | 17 ± 2 | 14 ± 2 | 19 ± 6 | 14 ± 2 |

***P* < 0.05; One-way ANOVA (with *Post hoc* Newman Keul’s test) in comparison to 3μM DHP

Table S13. The 3α-HSD inhibitor indomethacin (indo) reduces the effect of ganaxolone (Ganax) but not allopregnanolone (Allo) incubation treatment on GABA_A_R mIPSCs of layer 2/3 cortical neurones (P60-75) of WT mice

|  | **WT**  **Control**  **(n=35)** | **WT**  **30μM Indo**  **(n=6)** | **WT**  **100μM Indo**  **(n=7)** | **WT**  **100nM Allo**  **(n=9)** | **WT**  **100nM Allo**  **& 100μM Indo**  **(n=7)** | **WT**  **300nM Ganax**  **(n=10)** | **WT**  **300nM Ganax & 30μM Indo**  **(n=10)** | **WT**  **300nM Ganax & 100μM Indo**  **(n=8)** |
| --- | --- | --- | --- | --- | --- | --- | --- | --- |
| **Peak amp (pA)** | -59 ± 2 | -53 ± 2 | -53 ± 2 | -78 ± 2 | -77 ± 4 | -82 ± 6 | **-63 ± 3 | **-54 ± 4 |
| **Rise Time (ms)** | 0.4 ± 0.1 | 0.4 ± 0.1 | 0.4 ±0.1 | 0.5 ± 0.1 | 0.5 ± 0.1 | 0.5 ± 0.1 | 0.4 ± 0.1 | 0.5 ± 0.1 |
| **Charge transfer (fC)** | -242 ± 9 | -222 ± 6 | -235 ± 13.5 | -932 ± 84 | -915 ± 10 | -749 ± 88 | **-374 ± 39 | **-289 ± 30 |
| **T70 (ms)** | **5.2 ± 0.2** | **5.1 ± 0.2** | **5.8 ± 0.2** | **18.6 ± 1.4** | **17.0 ± 0.5** | **13.4 ± 1** | ****8.7 ± 0.7** | ****6.3 ± 0.3** |
| **Tau w (ms)** | **4.0 ± 0.1** | **4.6 ± 0.2** | **4.2 ± 0.1** | **12.5 ± 1** | **13.5 ± 0.8** | **10.9 ± 1** | ****6.2 ± 0.5** | ****5.4 ± 0.3** |
| **Frequency (Hz)** | 17 ± 2 | 14 ± 2 | 14 ± 2 | 15 ± 3 | 10 ± 2 | 20 ± 3 | 23 ± 2 | #9.3 ± 1 |

***P* < 0.05; One-way ANOVA (with *Post hoc* Newman Keul’s test) in comparison to 300nM ganaxolone

Table S14. The effect of intracellular allopregnanolone (Allo) on GABA_A_R mIPSCs of layer 2/3 cortical neurones (P60-75) of WT or *ob/ob* mice

|  | **WT**  **Control**  **(n=35)** | **WT**  **3μM Allo**  **(n=6)** | **WT**  **10μM Allo**  **(n=7)** | ***Ob/ob***  **(n=25)** | ***Ob/ob***  **3μM Allo**  **(n=7)** | ***Ob/ob***  **10μM Allo**  **(n=5)** |
| --- | --- | --- | --- | --- | --- | --- |
| **Peak amp (pA)** | -59 ± 2 | -64 ± 3 | **-95 ± 4 | -66 ± 2 | -61 ± 2 | **-79 ± 6 |
| **Rise Time (ms)** | 0.4 ± 0.1 | 0.5 ± 0.1 | 0.5 ± 0.1 | 0.4 ± 0.1 | 0.5 ± 0.1 | 0.5 ± 0.1 |
| **Charge transfer (fC)** | -242 ± 9 | -354 ± 49 | **-1389 ± 139 | -255 ± 12 | -342 ± 27 | **-1140 ± 173 |
| **T70 (ms)** | **5.2 ± 0.2** | ****7.7 ± 1** | ****22.3 ± 1.4** | **4.7 ± 0.1** | ****7.4 ± 0.5** | ****23.0 ± 2** |
| **Tau w (ms)** | **4.0 ± 0.1** | ****5.8 ± 0.8** | ****16.1 ± 1.3** | **3.5 ± 0.1** | ****5.7 ± 0.4** | ****15.5 ± 2** |
| **Frequency (Hz)** | 17 ± 2 | 22 ± 4 | 20 ± 3 | 16 ± 3 | 20 ± 4 | 14 ± 5 |

***P* < 0.05; One-way ANOVA, in comparison to strain-matched control

Table S15. The effect of intracellular ganaxolone (Ganax) on GABA_A_R mIPSCs of layer 2/3 cortical neurones (P60-75) of WT or *ob/ob* mice

|  | **WT**  **Control**  **(n=35)** | **WT**  **3μM Ganax**  **(n=6)** | **WT**  **10μM Ganax**  **(n=6)** | ***Ob/ob***  **(n=25)** | ***Ob/ob***  **3μM Ganax**  **(n=6)** | ***Ob/ob***  **10μM Ganax**  **(n=6)** |
| --- | --- | --- | --- | --- | --- | --- |
| **Peak amp (pA)** | -59 ± 2 | -63 ± 4 | -69 ± 6 | -66 ± 2 | -60 ± 2 | -66 ± 4 |
| **Rise Time (ms)** | 0.4 ± 0.1 | 0.4 ± 0.1 | 0.4 ± 0.1 | 0.4 ± 0.1 | 0.4 ± 0.1 | 0.4 ± 0.1 |
| **Charge transfer (fC)** | -242 ± 9 | **-347 ± 15 | **-420 ± 55 | -255 ± 12 | -302 ± 21 | **-401 ± 50 |
| **T70 (ms)** | **5.2 ± 0.2** | ****7.1 ± 0.4** | ****8.1 ± 0.6** | **4.7 ± 0.1** | ****6.9 ± 0.3** | ****8.2 ± 0.7** |
| **Tau w (ms)** | **4.0 ± 0.1** | ****5.5 ± 0.4** | ****6.1 ± 0.3** | **3.5 ± 0.1** | ****5.0 ± 0.3** | ****6.4 ± 0.6** |
| **Frequency (Hz)** | 17 ± 2 | 18 ± 4 | 16 ± 2 | 16 ± 3 | 12 ± 3 | 12 ± 1 |

***P* < 0.05; One-way ANOVA, in comparison to strain-matched control

Table S16. The effect of pipette-applied ganaxolone and indomethacin on GABA_A_R mIPSCs of layer 2/3 cortical neurones (P60-75) of WT or *ob/ob* mice

|  | **WT**  **Control**  **(n=35)** | **WT**  **100μM Indo**  **(n=5)** | **WT**  **10μM Ganax**  **(n=6)** | **WT**  **10μM Ganax**  **&100μM Indo**  **(n=4)** | ***Ob/ob***  **(n=25)** | ***Ob/ob***  **100μM Indo**  **(n=5)** | ***Ob/ob***  **10μM Ganax**  **(n=6)** | ***Ob/ob***  **10μM Ganax**  **&100μM Indo**  **(n=4)** |
| --- | --- | --- | --- | --- | --- | --- | --- | --- |
| **Peak amp (pA)** | -59 ± 2 | -75 ± 2 | -69 ± 6 | -72 ± 7 | -66 ± 2 | -75 ± 2 | -66 ± 4 | -72 ± 5 |
| **Rise Time (ms)** | 0.4 ± 0.1 | 0.4 ± 0.1 | 0.4 ± 0.1 | 0.5 ± 0.1 | 0.4 ± 0.1 | 0.4 ± 0.1 | 0.4 ± 0.1 | 0.5 ± 0.1 |
| **Charge transfer (fC)** | -242 ± 9 | -306 ± 28 | -420 ± 55 | -494 ± 68 | -255 ± 12 | -228 ± 22 | -401 ± 50 | -497 ± 39 |
| **T70 (ms)** | **5.2 ± 0.2** | **4.8 ± 0.2** | **8.1 ± 0.6** | **9.4 ± 0.5** | **4.7 ± 0.1** | **4.9 ± 0.3** | **8.2 ± 0.7** | **9.5 ± 0.3** |
| **Tau w (ms)** | **4.0 ± 0.1** | **3.9 ± 0.4** | **6.1 ± 0.3** | **7.3 ± 0.6** | **3.5 ± 0.1** | **3.7 ± 0.3** | **6.4 ± 0.6** | **6.9 ± 0.4** |
| **Frequency (Hz)** | 17 ± 2 | 16 ± 4 | 16 ± 2 | 14 ± 2 | 16 ± 3 | 19 ± 5 | 12 ± 1 | 14 ± 1 |

Table S17. The effect of DHP applied acutely via the intracellular or extracellular routes on GABA_A_R mIPSCs of layer 2/3 cortical neurones (P60-75) of WT mice

|  | **WT**  **Control**  **(n=35)** | **WT**  **3μM DHP**  **in pipette**  **(n = 6)** | **WT**  **10μM DHP**  **in pipette**  **(n = 6)** |
| --- | --- | --- | --- |
| **Peak amplitude (pA)** | -59 ± 2 | -55 ± 2 | -67 ± 4 |
| **Rise Time (ms)** | 0.4 ± 0.1 | 0.4 ± 0.1 | 0.4 ± 0.1 |
| **Charge transfer (fC)** | -242 ± 9 | -229 ± 14 | **-302 ± 12 |
| **T70 (ms)** | **5.2 ± 0.2** | **5.3 ± 0.2** | **5.5 ± 0.4** |
| **Tau w (ms)** | **4.0 ± 0.1** | **3.9 ± 0.1** | **4.3 ± 0.3** |
| **Frequency (Hz)** | 17 ± 2 | 22 ± 1 | 18 ± 2 |

***P* < 0.05; One-way ANOVA, in comparison to age-matched control

Table S18. ***ob/ob* mice exhibit prolonged tail withdrawal time (seconds) from noxious heat in comparison to WT mice**

| **Temperature** | **WT**  **(n=20)** | ***Ob/ob***  **(n=20)** |
| --- | --- | --- |
| **46 ^o^C** | 12.1 ± 1 | 14.1 ± 0.6 |
| **48 ^o^C** | 5.4 ± 0.8 | **7.5 ± 0.9 |
| **50 ^o^C** | 2.5 ± 0.2 | **5.0 ± 1 |

***P* < 0.05, Mann-Whitney Rank Sum test

Table S19. Ganaxolone increases tail withdrawal latency (seconds) to a noxious thermal stimulus (50 ^o^C) in WT mice.

| **Time** | **WT**  **Control**  **(n=10)** | **WT**  **3mg/kg Ganax**  **(n = 10)** | **WT**  **10mg/kg Ganax**  **(n=10)** | **WT**  **30mg/kg Ganax**  **(n = 10)** |
| --- | --- | --- | --- | --- |
| **Baseline** | 1.9 ± 0.3 | 1.1 ± 0.1 | 1.5 ± 0.1 | 2.8 ± 0.7 |
| **30 minutes** | 2.0 ± 0.1 | 1.5 ± 0.2 | **6.1 ± 1.8 | **6.0 ± 2 |
| **60 minutes** | 2.5 ± 0.2 | 2.1 ± 0.3 | **5.2 ± 1.7 | **8.8 ± 2 |
| **120 minutes** | 2.7 ± 0.3 | 3.8 ± 1.3 | **7.3 ± 2.1 | **11.5 ± 1.8 |

***P* < 0.05, Kruskall Wallis One-way ANOVA, on ranks

Table S20. High doses of ganaxolone reduce amount of time (seconds) that WT mice are able to remain on the rotarod

| **Time** | **WT**  **Control**  **(n=5)** | **WT**  **5mg/kg Ganax**  **(n = 5)** | **WT**  **10mg/kg Ganax**  **(n=5)** | **WT**  **30mg/kg Ganax**  **(n = 5)** |
| --- | --- | --- | --- | --- |
| **Baseline** | 215 ± 28 | 155 ± 16 | 216 ± 24 | 211 ± 40 |
| **15 minutes** | 208 ± 28 | 165 ± 33 | 136 ± 20 | **42 ± 33 |
| **30 minutes** | 243 ± 27 | 211 ± 28 | 161 ± 30 | **54 ± 41 |
| **60 minutes** | 182 ± 35 | 172 ± 32 | 196 ± 21 | **36 ± 26 |
| **120 minutes** | 208 ± 45 | 220 ± 17 | 221 ± 12 | 155 ± 65 |

***P* < 0.05, Kruskall Wallis One-way ANOVA, on ranks

Table S21. *ob/ob* mice exhibit greater response rate to von Frey Filaments in comparison to WT mice, in a manner consistent with mechanical hypersensitivity

| **Von Frey Filament** | **WT**  **(n=33)** | ***Ob/ob***  **(n=33)** |
| --- | --- | --- |
| **0.16 g** | 30.9 ± 3.2% | **41.8 ± 3.9% |
| **0.4 g** | 56.4 ± 3.7% | **68.8 ± 3.8% |
| **0.6 g** | 69.1 ± 3.5% | **85.2 ± 2.5% |
| **1.0 g** | 84.8 ± 2.9% | 92.7 ± 1.7% |

***P* < 0.05, Mann-Whitney Rank Sum test

Table S22. The β-CD vehicle had no impact on the response to any of the vF filaments in WT and *ob/ob* mice

| **Von Frey Filament** | **WT**  **Pre**  **β-CD vehicle**  **(n=10)** | **WT**  **Post**  **β-CD vehicle**  **(n=10)** | ***Ob/ob***  **Pre**  **β-CD vehicle**  **(n=10)** | ***Ob/ob***  **Post**  **β-CD vehicle**  **(n=10)** |
| --- | --- | --- | --- | --- |
| **0.16 g** | 36 ± 6% | 46 ± 5% | 43 ± 6% | 39 ± 5% |
| **0.4 g** | 70 ± 6% | 68 ± 7% | 63 ± 7% | 53 ± 5% |
| **0.6 g** | 77 ± 6% | 74 ± 6% | 83 ± 5% | 74 ± 5% |
| **1.0 g** | 90 ± 5% | 88 ± 6% | 89 ± 5% | 84 ± 4% |

Table S23. Ganaxolone reduces mechanical nociceptive pain in both WT mice, but not mechanical hypersensitivity

| **Von Frey Filament** | **WT**  **Pre**  **10 mg/kg Ganax**  **(n=10)** | **WT**  **Post**  **10 mg/kg Ganax**  **(n=10)** |
| --- | --- | --- |
| **0.16 g** | 31 ± 6% | 42 ± 6% |
| **0.4 g** | 61 ± 6% | 57 ± 7% |
| **0.6 g** | 79 ± 4% | 65 ± 6% |
| **1.0 g** | 94 ± 3% | **82 ± 5% |

***P* < 0.05, Wilcoxon signed rank test (before & after)

Table S24. Ganaxolone reduces mechanical hypersensitivity and mechanical nociceptive pain in *ob/ob* mice

| **Von Frey Filament** | ***Ob/ob***  **Pre**  **10 mg/kg Ganax**  **(n=10)** | ***Ob/ob***  **Post**  **10 mg/kg Ganax**  **(n=10)** |
| --- | --- | --- |
| **0.16 g** | 42 ± 7% | 38 ± 8% |
| **0.4 g** | 66 ± 7% | **49 ± 6% |
| **0.6 g** | 82 ± 5% | **59 ± 7% |
| **1.0 g** | 92 ± 4%, | **74 ± 8% |

***P* < 0.05, Wilcoxon signed rank test (before & after)
