# Supplementary material for: Neurosteroids are reduced in diabetic neuropathy and may be associated with the development of neuropathic pain
Source: F1000Res. 2016 Aug 5;5:1923. [Version 1] doi: 10.12688/f1000research.9034.1 (PMC5345788; doi:10.12688/f1000research.9034.1)
Supplement: Peer review history

# Peer review: Referee response for version 1

**DOI:** 10.5256/f1000research.9720.r20664

**Published:** 2017-03-03

**Authors:** Xue-Jun Song, Changyu Jiang

**Affiliations:** 1 Center for Anesthesiology and Pain Medicine, Department of Anesthesiology, Peking University Cancer Hospital and Institute, Beijing , China; 2 Nanshan Hospital, Guangdong, Shenzhen, China

In this manuscript, the author investigated the neural electrophysiological characterizations of the spinal cord, the nucleus reticularis of the thalamus and the cerebral cortex by means of whole-cell patch-clamp recordings. The results suggested that diabetic mice showed reduced neurosteroid tone but enhanced sensitivity to some neurosteroids, ob/ob mice exhibited mechanical hyperalgesia and allodynia, which was reduced by neurosteroids applied exogenously. The author concluded that the reduced endogenous neurosteroid tone in ob/ob mice may be linked to their hypersensitivity.

 Questions:

 1. The author concluded the loss of neurosteroid tone in ob/ob mice. There is no further mechanism study.

 2. What is the neurosteroid receptor expression in ob/ob mice, compared with WT mice? Is there any alternation in diabetic mice?

 3.   The author did not show the frequency of mIPSC in lamina II of the spinal dorsal horn, nRT and layer 2/3 of the cortex. How about the frequency of mIPSC in those sites with development. Please show some representative consecutive chart recording.

 4. According to the results, the amplitude of mIPSC is around 35-40 Pa, which is larger than our experience. Can the author give the reason.

 5. Please can the author check the alphabetical sequences of the figures and figure legends.

We have read this submission. We believe that we have an appropriate level of expertise to confirm that it is of an acceptable scientific standard, however we have significant reservations, as outlined above.

# Peer review: Referee response for version 1

**DOI:** 10.5256/f1000research.9720.r19599

**Published:** 2017-02-14

**Authors:** Anthony Dickenson

**Affiliations:** 1 Department of Neuroscience, Physiology and Pharmacology, University College London, London, UK

This is an interesting study using electrophysiological and behavioural approaches in strains of mice to gauge the potential changes in neurosteroid signalling in diabetic neuropathy. The study extends through the central nervous system and the data is suggestive of important roles of these substances. The work is detailed and meticulous. However, using *in vitro* slices cannot allow for investigation of identified neurons in terms of pain inputs and how these may be altered by neurosteroids or indeed, neuropathy.

I have read this submission. I believe that I have an appropriate level of expertise to confirm that it is of an acceptable scientific standard.

# Peer review: Referee response for version 1

**DOI:** 10.5256/f1000research.9720.r15694

**Published:** 2016-08-15

**Authors:** Christopher Connolly

**Affiliations:** 1 Centre for Environmental Change and Human Resilience (CECHR), University of Dundee, Dundee, UK

I am not an electrophysiologist by training but am an expert in GABA(A) receptors.

 Summary:

- TIID mice have reduced neurosteroid tone but increased sensitivity to some neurosteroids.
- Ob/ob mice have mechanical hyperalgesia and allodynia (reduced by neurosteroid application).

Conclusions:

- Reduced neurosteroid tone may be linked to hypersensitivity.
- Neurosteroids may exert analgesic effects by restoring GABAergic inhibitory tone.

 

 Comments:

1. I’m not clear on the comments relating to a developmental change in tone, yet the comment that this may be due to fluctuations in endogenous neurosteroid tone. I assume the authors do not mean to imply that development also fluctuates. Perhaps this needs rephrasing.
2. Is anything known about the loss of neurosteroid tone? Is it due to altered neurosteroid production/degradation or receptor expression to non-responsive receptors?
3. Presumably the use of the smaller cyclodextrins excludes the possibility of membrane cholesterol involvement. This should be stated.
4. Has the time required for neurosteroid diffusion in a slice been confirmed using isolated neurons in culture (where no diffusion barrier exists)? This would help distinguish between neurosteroid-induced chronic changes in protein trafficking/gene expression and genuine diffusion if the changes can occur more quickly in culture.
5. Does the lack of finasteride on WT and ob/ob mice indicate that there is no endogenous basal neurosteroid-induced tone? The half-life of neurosteroid should be stated so that it may be considered with regard to being relevant.
6. It is interesting that galaxalone might be an allosteric modulator of GABA(A)Rs (as the other possibility of indomethacin as an antagonist has been excluded).
7. The rotarod test seems unfair when comparing fat mice to WT, but is valid in determining the detrimental effects of high doses of neurosteroid.
8. The effects of ganaxolone on vF is encouraging to indicate  its potential relief from mechanical pain.
9. The possibility of differential binding sites to neurosteroids that could be differentially expressed is worthy of mention.
10. With the evidence presented here, I understand that the effects of Progesterone and DHP are greater in diabetic models, which would support an upregulation of the neurosteroid synthesis pathway as some compensatory mechanism. However, the evidence for the loss of neurosteroid tone remains elusive and probably upstream of the production of progesterone. This would presumably imply a deficit in mitochondrial cholesterol uptake, metabolism to pregnenolone, its efflux into the cytosol, or conversion to progesterone (from here on, the pathway to allopregnanolone is normal or enhanced). This needs to be spelled out more clearly.

I have read this submission. I believe that I have an appropriate level of expertise to confirm that it is of an acceptable scientific standard, however I have significant reservations, as outlined above.

# Response

**Published:** 2017-02-17

**Authors:** Stephen Humble

**Affiliations:** Imperial College London, UK

Authors Comments in response to the reviewer

 Thank you very much indeed for talking the time to review the paper

 1. The author agrees that development does not fluctuate. Neurosteroid tone starts out very high in the most immature neurons then decreases as with maturity. At around P20 the neurosteroid tone is almost negligible, but a modest neurosteroid tone has reappeared in mature neurones by P60 (Humble 2013, Brown 2012). The presence of the neurosteroid tone may be revealed with the use of cyclodextrin. Separately the overall τ _W _of the mIPCs also decreases independently of the neurosteroid tone with progressive maturity (Humble 2013, Brown 2012).

 2. Loss of neurosteroid tone in development thought to be due to reduced production. But separately there is also changes in the expression of different receptor subtypes (e.g. alpha-2 as per Bosman *et al.,* 2005).

 3. I can insert: ‘The use of small cyclodextrins was used to attempt to control for the generic effects of cyclodextrin such as an interaction with the cholesterol within the neuronal membrane.’

 4.The author had not performed experiments using isolated neurons in culture, nor explored specific potential changes in trafficking/gene expression. The lack of these experiments could be considered a relative limitation of the study and would be a useful complimentary study.  The author used different methods of  neurosteroid presentation in order to explore the issue of diffusion and ensure that neurosteroid penetration of the slice was optimal in each situation. In order to maximize the neurosteroid effect the slices were incubated more than an hour with the relevant neurosteroid compound as per work on other lipophilic GABAergic compounds by other authors (Benkwitz *et al.,* 2007; Gredell *et al.,* 2004)

 5. The lack of effect of finasteride on WT and ob/ob mice is not sufficient to make the statement that there is no endogenous basal neurosteroid-induced tone. Indeed, there is in fact an endogenous basal neurosteroid-induced tone as illustrated by the experiments with cyclodextrin. The lack of observed effect with finasteride is consistent with other authors (Brown, 2012 Thesis). The lack of effect with finasteride in this context is explained as follows: Finasteride inhibits the production of new neurosteroid via 5α-reductase inhibition. However it does not impact on the endogenous neurosteroid compounds that are already present within the slice preparation. This question was considered during the project and it was decided that the appropriate way to answer this question would be to inject finasteride in vivo, some hours/days prior to the in vitro experiments.  The half-life of the neurosteroid Allopregnanolone is approximately 30 minutes (Mellon et al., 2008). However, a functional equilibrium typically exists between Allopregnanlone and its neurosteroid precursors which act as an immediate reservoir that would not be blocked instantly by finasteride, which inhibits 5αR rather than 3α-HSD (Figure 1).

 6. It is very interesting

 7. Agreed. The rotarod test results for the ob/ob are included for completeness and for demonstrating the phenotype but have minimal validity other than that. The ob/ob rotarod experiments illustrate why the sedative effects of neurosteroid were studied in WT rather than the ob/ob. The ob/ob is type-2 diabetic because of obesity. Other obese mouse models such as the db/db also have type-2 diabetes. There does not appear to be an obese mouse model without type-2 diabetes. Thus the respective impacts of obesity and type-2 diabetes on rotarod ability cannot be differentiated.

 8. This is a potentially translatable finding.

 9. It is known that general affinity for the GABAA receptor varies between different neurosteroid compounds. Separately, GABAA receptors expressing specific subunits have greater affinity for a given neurosteroid. Indeed, the following is an extract from my thesis:

 ‘THIP is a low affinity partial agonist at receptors expressing the g subunit ( *i.e.* most GABA _A_Rs), but has high affinity for GABA _A_Rs that express the a4b3d subunits where it behaves as a ‘superagonist’ compared to GABA (Brown *et al.,* 2002; Farrant & Nusser, 2005; Krogsgaard-Larsen *et al.,* 2004). d-GABA _A_Rs are predominantly located at extrasynaptic locations; therefore THIP is a useful pharmacological agent for the selective activation of tonic currents (Belelli *et al.,* 2005; Farrant & Nusser, 2005).’  (Humble, 2013)

 For reasons of brevity the issue of differential binding sites to neurosteroids was not explored significantly within the paper, but this was covered in substantial detail within my thesis (Humble, 2013). It is summarised in the Conclusions section of the thesis:

 ‘Pipette-applied neurosteroids allopregnanolone and ganaxolone (but not DHP) were also able to prolong the decay time of cortical GABA _A_R mIPSCs in a differential manner.  Allopregnanolone induced a greater effect than ganaxolone on GABA _A_R mIPSC decay time, but there was no difference in the response to the respective drugs between the WT and *ob/ob* mice.  These findings suggest that neurosteroids are able to modulate the GABA _A_R from the intracellular compartment and that the sensitivity of the GABA _A_R is the same for WT and *ob/ob* mice.  In addition, when indometacin was co-applied in the pipette with ganaxolone it had no impact on the efficacy of ganaxolone.  This observation is inconsistent with the notion that indometacin competes with ganaxolone for its binding of the GABA _A_R.’ (Humble, 2013)

 10. This is an excellent point and is the subject of a Brief Communication that I have written on the subject of Mitochondrial dysfunction and neurosteroid synthesis in diabetic neuropathy that will be published shortly.  I would be happy to refer to this further in the discussion.
